# N^6^‐methyladenosine‐modified lncRNA ARHGAP5‐AS1 stabilises CSDE1 and coordinates oncogenic RNA regulons in hepatocellular carcinoma

**DOI:** 10.1002/ctm2.1107

**Published:** 2022-11-10

**Authors:** Jiandong Liu, Nasha Zhang, Jiajia Zeng, Teng Wang, Yue Shen, Chi Ma, Ming Yang

**Affiliations:** ^1^ Shandong Provincial Key Laboratory of Radiation Oncology Cancer Research Center Shandong Cancer Hospital and Institute Shandong First Medical University and Shandong Academy of Medical Sciences Jinan Shandong Province China; ^2^ Department of Radiation Oncology Shandong Cancer Hospital and Institute Shandong First Medical University and Shandong Academy of Medical Sciences Jinan Shandong Province China; ^3^ Jiangsu Key Lab of Cancer Biomarkers, Prevention and Treatment Collaborative Innovation Center for Cancer Personalized Medicine Nanjing Medical University Nanjing Jiangsu Province China; ^4^ Shandong University Cancer Center Jinan Shandong Province China

**Keywords:** ARHGAP5‐AS1, *N* ^6^‐methyladenosine, lncRNA, CSDE1, hepatocellular carcinoma

## Abstract

**Background:**

Hepatocellular carcinoma (HCC) ranks fourth among the malignancies leading to cancer‐related deaths all around the world. It is increasingly evident that long non‐coding RNAs (lncRNAs) are a key mode of hepatocarcinogenesis. As the most prevalent mRNA modification form, *N* ^6^‐methyladenosine (m^6^A) regulates gene expression by impacting multiple aspects of mRNA metabolism. However, there are still no reports on genome‐wide screening and functional annotation of m^6^A‐methylated lncRNAs in HCC.

**Methods:**

The m^6^A modification and biologic functions of ARHGAP5‐AS1 in HCC were investigated through a series of biochemical assays. Clinical implications of ARHGAP5‐AS1 were examined in tissues from HCC patients.

**Results:**

After systematically analysing the m^6^A‐seq data of HCC cells, we identified 22 candidate lncRNAs with evidently dysregulated m^6^A levels. Among these lncRNAs, we found that ARHGAP5‐AS1 is the lncRNA with the highest levels of m^6^A modification and significantly increased expression in HCC specimens. METTL14 acts as the m^6^A writer of ARHGAP5‐AS1 and IGF2BP2 stabilises the lncRNA as its m^6^A reader. ARHGAP5‐AS1 remarkably promotes malignant behaviours of HCC cells ex vivo and in vivo. We identified oncoprotein CSDE1 working as the interacting protein of the lncRNA and TRIM28 as the E3 ligase of CSDE1 in HCC. Interestingly, ARHGAP5‐AS1 could attenuate interactions between CSDE1 and TRIM28, which prevents the degradation of CSDE1 via the ubiquitin‐proteasome pathway. Elevated levels of CSDE1 coordinate oncogenic RNA regulons, promote translation of *VIM* and *RAC1* and activate the ERK pathway, which contributes to HCC prognosis.

**Conclusions:**

Our study reveals a new paradigm in m^6^A‐modified lncRNAs controlling CSDE1‐mediated oncogenic RNA regulons and highlights lncRNAs as potential targets for future therapeutics against HCC.

## Graphical abstract

- ARHGAP5‐AS1 is the lncRNA with high m6A levels and significantly increased expression in HCC.
- METTL14 is the m6A writer of ARHGAP5‐AS1 and IGF2BP2 is its m6A reader.
- Oncogenic ARHGAP5‐AS1 attenuates interactions between CSDE1 and TRIM28, which prevents CSDE1 degradation via the proteasome.
- CSDE1 coordinates oncogenic RNA regulons and activates the ERK pathway, which contributes to HCC prognosis.

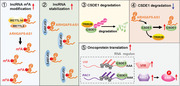

## 1 BACKGROUND

Hepatocellular carcinoma (HCC) is the sixth lethal malignancy and ranks fourth among neoplasms leading to cancer‐related deaths all around the world.^1^, ^2^, ^3^ Considerable global differences in the morbidity and mortality of HCC exist and about 85% of HCC patients are diagnosed in Eastern Asia as well as North Africa.^1^, ^2^, ^3^ Regrettably, the 5‐year survival rate of HCC patients is only 18%.^1^, ^2^, ^3^ Besides multiple well‐established risk factors, such as infection of hepatitis B virus (HBV) and/or hepatitis C virus (HCV), aflatoxin B1 intakes, heavy cigarette smoking and excessive alcohol consumption,^1^, ^2^ the importance of long non‐coding RNAs (lncRNAs) as a key, regulatory mode of hepatocarcinogenesis is increasingly evident.^4^, ^5^, ^6^, ^7^, ^8^, ^9^

Accumulated evidences demonstrated that *N* ^6^‐methyladenosine (m^6^A) plays important, wide‐ranging roles in various malignancies including HCC via post‐transcriptionally regulating gene expression.^10^, ^11^, ^12^ As a chemical derivative of adenosine in RNA, m^6^A shows a frequency of 0.15%‐0.6% of all adenosines across the mammal transcriptome.^10^, ^11^, ^12^ Typically, the m^6^A methylation is deposited onto transcripts of mRNAs, lncRNAs and primary microRNAs (pri‐miRNAs) by the METTL3/METTL14 methyltransferase complex co‐transcriptionally.^10^, ^11^, ^12^ In human cells, METTL14 interacts with METTL3 and acts the key methyltransferase to convert A to m^6^A in RNAs. Genetic knockout of *Mettl14* is developmentally lethal in mice, indicating its crucial role in numerous physiological and pathophysiological processes via regulating m^6^A modification.^13^, ^14^ Although several mRNAs have been identified as targets of METTL14‐induced m^6^A modification,^15^, ^16^, ^17^ it is still largely unclear how m^6^A‐modified lncRNAs controlled by METTL14 contribute to HCC development.

In this study, we firstly recognized 22 candidate lncRNAs with evidently dysregulated m^6^A levels after systematically analysing the m^6^A‐seq data of HCC cells with or without silencing of *METTL14*. Among these lncRNAs, we found that ARHGAP5‐AS1 is the lncRNA with the highest levels of m^6^A modification and increased expression in HCC specimens. METTL14 acts as the m^6^A writer of ARHGAP5‐AS1 and IGF2BP2 as its m^6^A reader to stabilise lncRNA ARHGAP5‐AS1 in HCC. LncRNA ARHGAP5‐AS1 remarkably promotes malignant behaviours of HCC cells ex vivo and in vivo. Interestingly, ARHGAP5‐AS1 attenuates interactions between the oncoprotein CSDE1 and its E3 ligase TRIM28, which prevents CSDE1 degradation via the proteasome. Particularly, elevated levels of CSDE1 promote the translation and expression of *VIM* and *RAC1* genes and, thus, HCC cancerous traits.

## 2 METHODS

### 2.1 Identification of candidate lncRNAs

In order to determine m^6^A‐modified lncRNAs in HCC, we thoroughly inspected the HepG2 cell m^6^A‐seq profiles after knocking‐down expression of *METTL14* or not (GSE90642).^15^ Among 1,130 genes with dysregulated m^6^A modification levels (m^6^A fold change <0.667 or >1.5) after silencing of *METTL14*,^15^ there were 22 lncRNAs (*ARHGAP5‐AS1*, *STX16‐NPEPL1*, *TUG1*, *ENTPD1‐AS1*, *FAM157A*, *MIR570HG*, *THAP9‐AS1*, *COX10‐AS1*, *CYTOR*, *ABALON*, *DHRS4‐AS1*, *LINC01146*, *MIR663AHG*, *C1QTNF1‐AS1*, *NDUFB2‐AS1*, *TSPEAR‐AS1*, *SLCO4A1‐AS1*, *DARS‐AS1*, *MZF1‐AS1*, *TEN1‐CDK3*, *MIR22HG* and *USP27X‐AS1*) with remarkably differential m^6^A modification in HepG2 cells.

### 2.2 Cell culture

Human HCC HepG2 and SK‐HEP‐1 cells were obtained from the cell bank of type culture, Chinese Academy of Sciences (Shanghai). HEK293T cell line is a generous gift from Dr. Yunshan Wang working at Jinan Central Hospital (Shandong Province, China). DMEM medium (Gibco, C11995500BT) with 10% foetal bovine serum (FBS; Gibco, 1347575) was used for the cultivation of all cell lines. All cells were examined mycoplasma negative once in a while.

### 2.3 RNA immunoprecipitation (RIP) and m^6^A RNA immunoprecipitation (MeRIP) assays

All RIP assays were operated with the Magna RIP RNA‐Binding Protein Immunoprecipitation Kit (Millipore, 17‐700) and the antibodies of IGF2BP1, IGF2BP2, IGF2BP3 or CSDE1 as well as IgG Isotype‐control (Table S1). The MeRIP assay was carried out using the same Kit (Millipore, 17‐700) with the m^6^A antibody or IgG Isotype‐control (Table S1). The target protein‐RNA complexes were then enriched with Dynabeads^®^ Protein G (Invitrogen, 10003D). Levels of various lncRNAs in the protein‐RNA complexes were detected by quantitative reverse transcription PCR (RT‐qPCR).

### 2.4 RT‐qPCR

Trizol reagent (Invitrogen, 94402) was used for the extraction of total RNAs. RNA samples were reverse‐transcribed into cDNAs with PrimeScript^TM^ RT Master Mix (TaKaRa, RR036A). The relative expression of eight lncRNAs (ARHGAP5‐AS1, LINC00152, C1QTNF1‐AS1, LINC00969, USP27X‐AS1, NDUFB2‐AS1, TEN1‐CDK3 and ABALON), *METTL14*, *IGF2BP2*, *S14*, *U2*, *GAPDH* and *CSDE1* were detected at least in triplicate with indicated primers (Table S2). The melting‐curve analyses were done to confirm PCR product specificity.

### 2.5 The expression, mutant and shRNA constructs of ARHGAP5‐AS1 and CSDE1

The human *ARHGAP5‐AS1* cDNA (NR_027263.1) with a tag sequence (5’‐GTCGTATCCAGTGCGAATACCTCGGACCCTGCACTGGATACGAC‐3’) at the RNA 3’‐end was synthesised and cloned into pcDNA3.1 by Genewiz (Suzhou, China), which was named as WT. Mutants 1, 2 and 3 are plasmids with the A‐to‐G mutation at the 876, 890 or 928 base of WT. The full‐length *ARHGAP5‐AS1* cDNA was also cloned into pCDH‐CMV‐MCS‐EF1α‐Puro. As a result, the resultant plasmid was designated A‐AS1. The full‐length *ARHGAP5‐AS1* cDNA with inserted T7 promoter upstream and downstream from the cloning site was also cloned into pcDNA3.1. The resultant plasmid was designated pcDNA‐A‐AS1. Two *ARHGAP5‐AS1* shRNAs (shA‐AS1‐1 or shA‐AS1‐2, respectively) or the negative control shRNA (shNC) (Table S3) were synthesised and cloned into pLKO.1 by Genewiz (Table S3). These plasmids were named shA‐AS1‐1, shA‐AS1‐2 or shNC. The cDNA for the HA‐tagged *CSDE1* (NM_007158.6) and truncated versions of HA‐tagged *CSDE1* were cloned into pcDNA3.1 (Genewiz, China). To guarantee the orientation and integrity of plasmids, Sanger sequencing was performed.

### 2.6 Cell transfection

Small interfering RNA (siRNA) duplexes for *METTL14*, *IGF2BP2*, *CSDE1*, *TRIM28* or *HERC5* and the negative control RNA duplex (NC) were all synthesised by Genepharma (Shanghai, China) and details are in Table S3. Transfection of all small RNAs was using INTERFERin reagent (Polyplus, 409–10), as reported previously.^18^, ^19^ The jetPRIME reagent (Polyplus, 114‐07) was used for transfection of all plasmids as reported previously.^19^

### 2.7 Western blot

Western blot was done with indicated antibodies (Table S1) as reported previously.^19^, ^20^ The ECL Western Blotting Substrate (Pierce, 32106) was used to visualise candidate proteins.

### 2.8 Patients and tissue specimens

Eighty‐five HCC patients (Shandong cohort, *n* = 26, and Jiangsu cohort, *n* = 59) were recruited between April 2009 and December 2016 in this study. The demographics and clinical characteristics of all HCC cases were previously reported.^18^, ^21^, ^22^ All HCC patients were Han Chinese. This study was approved by the Institutional Review Board of Shandong Cancer Hospital and Institute. Before enrolling on this study, every patient agreed and signed the informed consent. All experimental methods comply with the Helsinki Declaration and are carried out according to the approved guidelines.

### 2.9 Lentiviral transduction

To prepare recombinant lentiviral particles, HEK293T cells were transiently transfected with the psPAX2 (Addgene, #12260) and pMD2.G (Addgene, #12259) plasmids plus the A‐AS1, shA‐AS1‐1 or shA‐AS1‐2 plasmid. At 48 and 72 h after transfection, cell culture supernatants with recombinant lentiviral particles were collected and filtered. Human HepG2 and SK‐HEP‐1 cells were infected with various viral supernatant supplemented with 5μg/mL polybrene and selected with 2 mg/mL puromycin. LncRNA ARHGAP5‐AS1 expression levels in these infected HCC cells were then detected.

### 2.10 Cell proliferation analyses

For the stable *ARHGAP5‐AS1*‐OE or *ARHGAP5‐AS1*‐KD HepG2 or SK‐HEP‐1 cells, 3 × 10^4^ cells per well were seeded in 12‐well plates, harvested, and counted at indicated time points after seeding. A total of 1 × 10^4^ HepG2 or SK‐HEP‐1 cells were seeded and then transfected with 20 nmol/L *CSDE1* siRNAs (siC1‐1 and siC1‐2) or NC RNA. Transiently transfected HCC cells were counted at indicated time points after transfection as reported previously.^23^

### 2.11 Colony formation assays

A 6‐well plate was used to seed with a total of 1,000 stable *ARHGAP5‐AS1*‐OE or *ARHGAP5‐AS1*‐KD HepG2 or SK‐HEP‐1 cells per well. A total of 1,000 HepG2 or SK‐HEP‐1 cells per well were seeded into a 6‐well plate. After seeding, the cells were transfected with 20 nmol/L NC RNA, siC1‐1 or siC1‐2. After 14 days, HCC cell colonies in each well were dyed and counted.

### 2.12 Xenograft assays

To examine the effect of lncRNA *ARHGAP5‐AS1* in vivo, a total of 1 × 10^7^ stable *ARHGAP5‐AS1*‐KD (shNC, shA‐AS1‐1 or shA‐AS1‐2) SK‐HEP‐1 cells were subcutaneously inoculated into fossa axillaries of female nude BALB/c mice (five‐week‐old, Vital River Laboratory, Beijing, China). Tumour growth was monitored every 5 days when tumour volumes reached or were greater than 30 mm^3^. In in vivo metastasis assays, 2 × 10^6^ SK‐HEP‐1 cells with stable firefly luciferase expression and *ARHGAP5‐AS1*‐KD (shNC, shA‐AS1‐1 or shA‐AS1‐2) were injected into female nude mice from tail vein (*n* = 3 per group). Distant metastases of HCC cells were visualised by the IVIS Spectrum In Vivo Imaging System from PerkinElmer. Processes during all mice assays were approved by the Animal Care Committee of Shandong Cancer Hospital and Institute.

### 2.13 Wound healing assays and transwell assays for HCC

When the cell layer of HCC cells was almost confluent, straight wounds of the same width were scratched with a 10μl pipette tip. The wound closure rate was then quantified at unified time points. The transwell assays were performed as reported previously.^19^, ^20^ After 36 or 24 h, HepG2 or SK‐HEP‐1 cells migrated to the lower wells were stained and the number of migrated cells was counted.

### 2.14 Subcellular fractionation

The nuclear/cytoplasmic Isolation Kit (Biovision, P0028) was applied to separately isolate the cytoplasm fractions and nuclear fractions of HCC cells in accordance with the manufacturer's specification.

### 2.15 RNA pulldown

The RNA pulldown experiment was performed as reported previously.^20^ The pcDNA‐A‐AS1 plasmid was used as the template for ex vivo synthesis of lncRNA ARHGAP5‐AS1. Sense and antisense ARHGAP5‐AS1 RNAs were biotinylated and incubated together with HepG2 cell extracts and Streptavidin magnetic beads (Thermo Fisher, 88816). The pull‐downed proteins were then screened by liquid chromatography‐tandem mass spectrometry (LS‐MS/MS) (Hoogen Biotech Co., Shanghai, China) and verified by Western Blot.

### 2.16 Turnover assays

As reported previously, the turnover assays were performed.^23^ In brief, HepG2 and SK‐HEP‐1 cells with stable *ARHGAP5‐AS1*‐KD or *ARHGAP5‐AS1*‐OE were treated with cycloheximide (CHX) (MedChemExpress, HY‐12320/CS‐4985) to pause de novo protein synthesis. The protein levels of CSDE1 and GAPDH were then examined in HCC cells which were treated with CHX.

### 2.17 Ubiquitination assays

As reported previously, the ubiquitination assays were performed in HepG2 and SK‐HEP‐1 cells transfected with pcDNA3.1‐HA‐ubiquitin (HA‐Ub).^23^ Proteins in HCC cells treated with MG132 were immunoprecipitated to isolate ubiquitinated CSDE1 and then measured using the anti‐HA antibody.

### 2.18 Immunoprecipitation‐mass spectrometry (IP‐MS) and Co‐IP

To identify the potential E3 ubiquitin ligase(s) of CSDE1, IP‐MS was performed using the antibody of CSDE1. Co‐IP was performed between CSDE1 and TRIM28 as reported previously.^19^ In brief, HCC cells were lysed and then incubated with antibodies of CSDE1, TRIM28 or IgG (Invitrogen) (Table S1) at 4°C overnight. On the next day, cell lysates were incubated with Dynabeads^®^ Protein G beads (Invitrogen) and then washed with the NP‐40 lysis buffer, and then examined by LS‐MS/MS (Hoogen Biotech Co., Shanghai, China) or Western Blot.

### 2.19 Immunofluorescence and RNA FISH

The immunofluorescence assay was performed as previously reported.^20^ After permeabilisation and blockage, HepG2 cells were incubated with primary antibodies overnight. Cells were then stained with secondary antibodies (Table S1), washed with PBS and incubated with 4,6‐diamidino‐2‐phenylindole (DAPI). RNA FISH was performed to examine the co‐localization of lncRNA *ARHGAP5‐AS1* and CSDE1 protein using the lncRNA FISH Kit (RiboBio) and immunofluorescence staining of CSDE1 antibody. In short, cells were permeabilised with 0.5% Triton X‐100 and hybridised with the FISH probes overnight at 37 °C in dark. LncRNA *ARHGAP5‐AS1* signals were detected using Cy3 channels. CSDE1 was stained with its antibody and CoraLite488‐conjugated Goat Anti‐Rabbit IgG(H + L) (Table S1). A Zeiss LSM800 confocal microscope (Zeiss, Germany) was used to visualise images.

### 2.20 Statistics

Student's *t*‐test was performed to calculate the difference between the two groups. Spearman's correlation was utilised to calculate the significance of expression association between different genes. Kaplan–Meier plots and the log‐rank test were applied to examine the impacts of lncRNA ARHGAP5‐AS1 expression on HCC patients’ survival. A *p* value of less than 0.05 was considered statistical significance. SPSS software package (Version 16.0, SPSS Inc.) or GraphPad Prism (Version 5, GraphPad Software, Inc.) was used for all analyses.

## 3 RESULTS

### 3.1 m^6^A‐modified lncRNA ARHGAP5‐AS1 controlled by the m^6^A writer METTL14 and the m^6^A reader IGF2BP2

**FIGURE 1:**
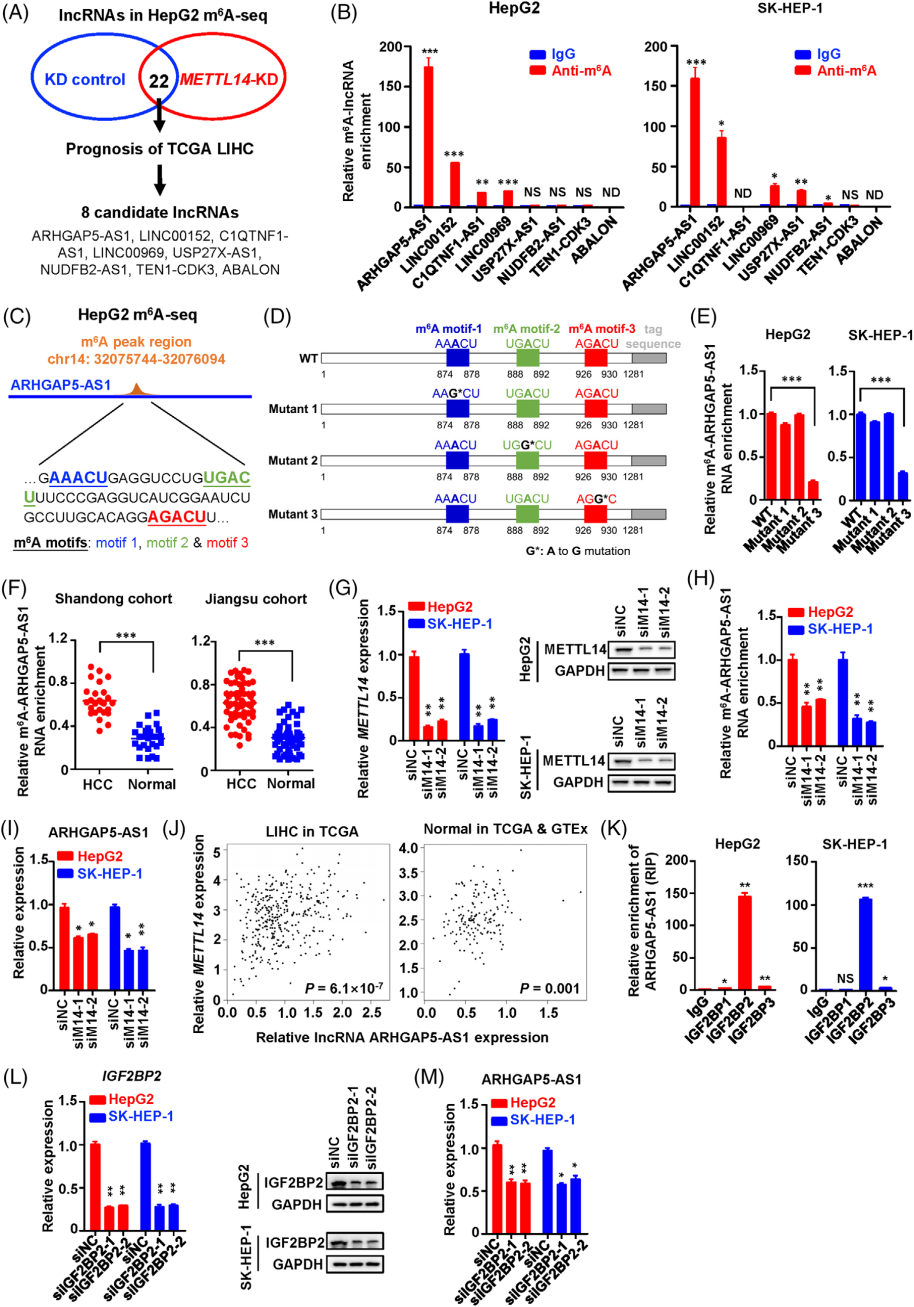
ARHGAP5‐AS1 is an m^6^A‐modified lncRNA controlled by the m^6^A writer METTL14 and the m^6^A reader IGF2BP2. (A) A flowchart of genome‐wide identification m^6^A‐modified lncRNAs in HCC. (B) The m^6^A RIP assays validated the m^6^A modification levels of these candidate lncRNAs in HepG2 and SK‐HEP‐1 cells. (C) The m^6^A peak in HepG2 and three potential m^6^A sites (876A, 890A and 928A) of ARHGAP5‐AS1 predicted by the SRAMP algorithm (http://www.cuilab.cn/sramp). (D) Schematic diagram of human full‐length *ARHGAP5‐AS1* with a tag sequence and its three mutated forms used in m^6^A RIP assays. (E) The m^6^A RIP assays showed that the ARHGAP5‐AS1 928A is its key m^6^A site in HCC cells. (F) The m^6^A‐ARHGAP5‐AS1 RNA levels in HCC tumours are significantly higher than those in the normal tissues. (G) *METTL14* was significantly knockdown using siM14‐1 or siM14‐2 in HCC cells. (H,I) The m^6^A RIP assays showed that silencing of *METTL14* evidently decreased m^6^A modification levels and expression levels of lncRNA ARHGAP5‐AS1. (J) Significant expression correlations exist between *METTL14* and ARHGAP5‐AS1 in TCGA HCC tissues and normal liver tissues. (K) The RIP assays indicated that IGF2BP2 is the reader protein with the highest binding affinity with lncRNA ARHGAP5‐AS1 in HCC cells. (L,M) Knock‐down of *IGF2BP2* markedly downregulated ARHGAP5‐AS1expression in HCC cells. Data information: Each value represents mean ± SD. The difference between the two groups was calculated using Student's *t*‐test. The significance of the association between lncRNA ARHGAP5‐AS1 expression and *METTL14* expression was calculated using Spearman's correlation. *p < 0.05; **p < 0.01; ***p < 0.001, NS, not significant, ND, not detectable. Data shows one representative of three independent experiments with three biological replicates.

To identify lncRNAs modified by m^6^A in HCC progression, we systematically analysed the m^6^A‐seq data of HepG2 cells with or without silencing of *METTL14* (Figure 1A).^15^ There were 22 lncRNAs with significantly differential m^6^A modification in HepG2 cells with or without silencing of *METTL14*. Among these lncRNAs, levels of eight lncRNAs (ARHGAP5‐AS1, LINC00152, C1QTNF1‐AS1, LINC00969, USP27X‐AS1, NDUFB2‐AS1, TEN1‐CDK3 and ABALON) are markedly associated with the prognosis of TCGA liver cancer (LIHC) patients (Table S4). We then validated the m^6^A modification levels of these candidate lncRNAs in HCC cells (Figure 1B). The m^6^A RIP assays indicated that ARHGAP5‐AS1 is the lncRNA with the highest levels of m^6^A modification in HCC cells. By using m^6^A‐seq in HepG2 and the SRAMP algorithm (http://www.cuilab.cn/sramp), we identified three potential m^6^A sites (876A, 890A and 928A) of ARHGAP5‐AS1 RNA (Figure 1C). Subsequent m^6^A‐specific RIP coupled RT‐qPCR analyses indicated that m^6^A levels of ARHGAP5‐AS1 RNA were significantly decreased in cells with ectopic expression of the ARHGAP5‐AS1 mutant 3 compared with cells with ectopic WT ARHGAP5‐AS1 expression (Figure 1D,E), suggesting that ARHGAP5‐AS1 928A is its key m^6^A site in HCC. We next investigated the m^6^A‐ARHGAP5‐AS1 RNA levels in our HCC patient cohorts and found that tumours had significantly higher m^6^A‐ARHGAP5‐AS1 RNA levels than the normal tissues (Figure 1F). After silencing of METTL14 (siM14‐1 or siM14‐2) in cells (Figure 1G), we observed evidently decreased m^6^A modification levels and expression levels of ARHGAP5‐AS1 (Figure 1H,I). In line with this, there are significant expression correlations between *METTL14* and ARHGAP5‐AS1 in HCC tissues (LIHC tissues of TCGA, *p* = 6.1 × 10^−7^) and normal liver tissues (TCGA and GTEx, *p* = .001) (Figure 1J).

It has been reported that the m^6^A readers IGF2BPs (IGF2BP1/2/3) stabilise their target RNAs in an m^6^A‐dependent way. Considering decreased m^6^A modification levels of ARHGAP5‐AS1 downregulates expression of the lncRNA, we examined whether IGF2BPs are readers of the m^6^A‐modified ARHGAP5‐AS1 in HCC cells. The RIP‐qPCR assays indicated that IGF2BP2 is the reader protein with the highest binding affinity with lncRNA ARHGAP5‐AS1 in HCC cells (both *p* < 0.01) (Figure 1K). Importantly, silencing of *IGF2BP2* markedly downregulated endogenous levels of ARHGAP5‐AS1 in HCC cells (all *p* < 0.05) (Figure 1L,M). Taken together, these data elucidated that METTL14 acts as the m^6^A writer of ARHGAP5‐AS1 and IGF2BP2 as its m^6^A reader to stabilise lncRNA ARHGAP5‐AS1 in HCC cells.

### 3.2 Identification of ARHGAP5‐AS1 as a novel oncogenic lncRNA in HCC

**FIGURE 2:**
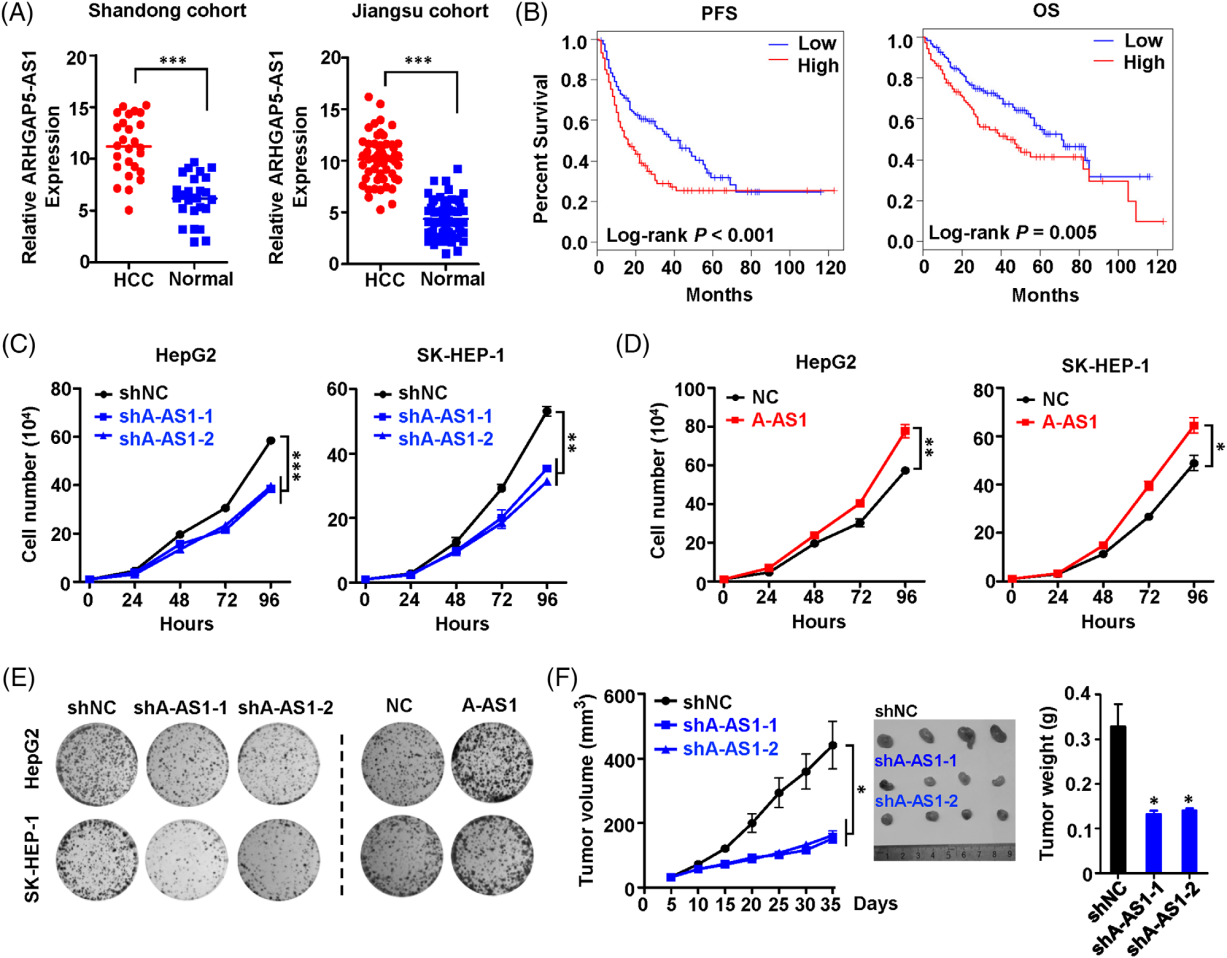
ARHGAP5‐AS1 promotes HCC cell proliferation in vitro and in vivo. (A) Expression levels of lncRNA ARHGAP5‐AS1 were measured using RT‐qPCR in tumour‐normal pairs of the Shandong cohort and Jiangsu cohort, respectively. All data of lncRNA ARHGAP5‐AS1 expression were normalised to *GAPDH* expression levels. (B) Kaplan‐Meier plots of HCC patients’ survival time were stratified according to ARHGAP5‐AS1 expression levels according to TCGA. (C,D) In HCC cells, silencing of *ARHGAP5‐AS1* resulted in obviously suppressed cell proliferation compared with the control cells. Stably *ARHGAP5‐AS1* overexpression could significantly enhance the proliferation of HCC cells. (E) Colony formation assays indicated that ARHGAP5‐AS1 significantly stimulated the clonogenicity of HCC cells. (F) Knockdown of *ARHGAP5‐AS1* significantly inhibited the growth of HCC xenografts compared with control xenografts after 35 days. Data information: Each value represents mean ± SD. The difference between the two groups was calculated using Student's *t*‐test. *p < 0.05; **p < 0.01, ***p < 0.001. Data shows one representative of three independent experiments with three biological replicates.

To explore the involvement of *ARHGAP5‐AS1* in hepatocarcinogenesis, we firstly detected its levels in HCC specimens and paired normal tissues of the Shandong cohort and Jiangsu cohort (Figure 2A). There was an obvious up‐regulation of lncRNA ARHGAP5‐AS1 in HCC tissues compared with that in normal liver specimens in the Shandong cohort or Jiangsu cohort (both *p* < 0.001) (Figure 2A and Table S5). In multiple independent HCC cohorts of Chinese (GSE84005 and GSE115018), Japanese (GSE17856) and Italian (GSE55092), lncRNA ARHGAP5‐AS1 levels expressed in cancerous tissues were consistently elevated, compared to normal specimens (all *p* < 0.05) (Figure S1A).^24^, ^25^, ^26^ Importantly, high ARHGAP5‐AS1 levels in HCC specimens were also correlated with shortened time of progression free survival (PFS) (Log‐rank *p* = 0.001) or overall survival (OS) (Log‐rank *p* = 0.005) (Figure 2B). Collectively, these findings demonstrated that lncRNA ARHGAP5‐AS1 may be a novel oncogene in HCC.

To reveal the biological significance of ARHGAP5‐AS1 in HCC, we developed the stable *ARHGAP5‐AS1*‐KD HepG2 and SK‐HEP‐1 cells (shA‐AS1‐1 and shA‐AS1‐2) and the stably *ARHGAP5‐AS1*‐OE HCC cells (A‐AS1) (Figure S1B). As shown in Figure 2C, stable *ARHGAP5‐AS1*‐KD resulted in an obviously inhibited proliferation of HCC cell lines compared to controls (*p* < .001). Stable *ARHGAP5‐AS1*‐OE could significantly enhance HCC cell proliferation (*p* < 0.001) (Figure 2D). Colony formation results also supported the oncogene role of lncRNA ARHGAP5‐AS1 in HCC (Figure 2E and Figure S1C). We then examined the oncogenic functions of ARHGAP5‐AS1 in vivo. We found that the *ARHGAP5‐AS1*‐KD HCC xenografts grew markedly slow as compared with the control xenografts (both *p* < 0.05) (Figure 2F). There were also obviously decreased tumour weights in the *ARHGAP5‐AS*1‐KD group compared to the control group (Figure 2F), which is in support of the oncogenic role of ARHGAP5‐AS1 in HCC.

### 3.3 LncRNA ARHGAP5‐AS1 promoted invasiveness of HCC cells and their metastases in mice

**FIGURE 3:**
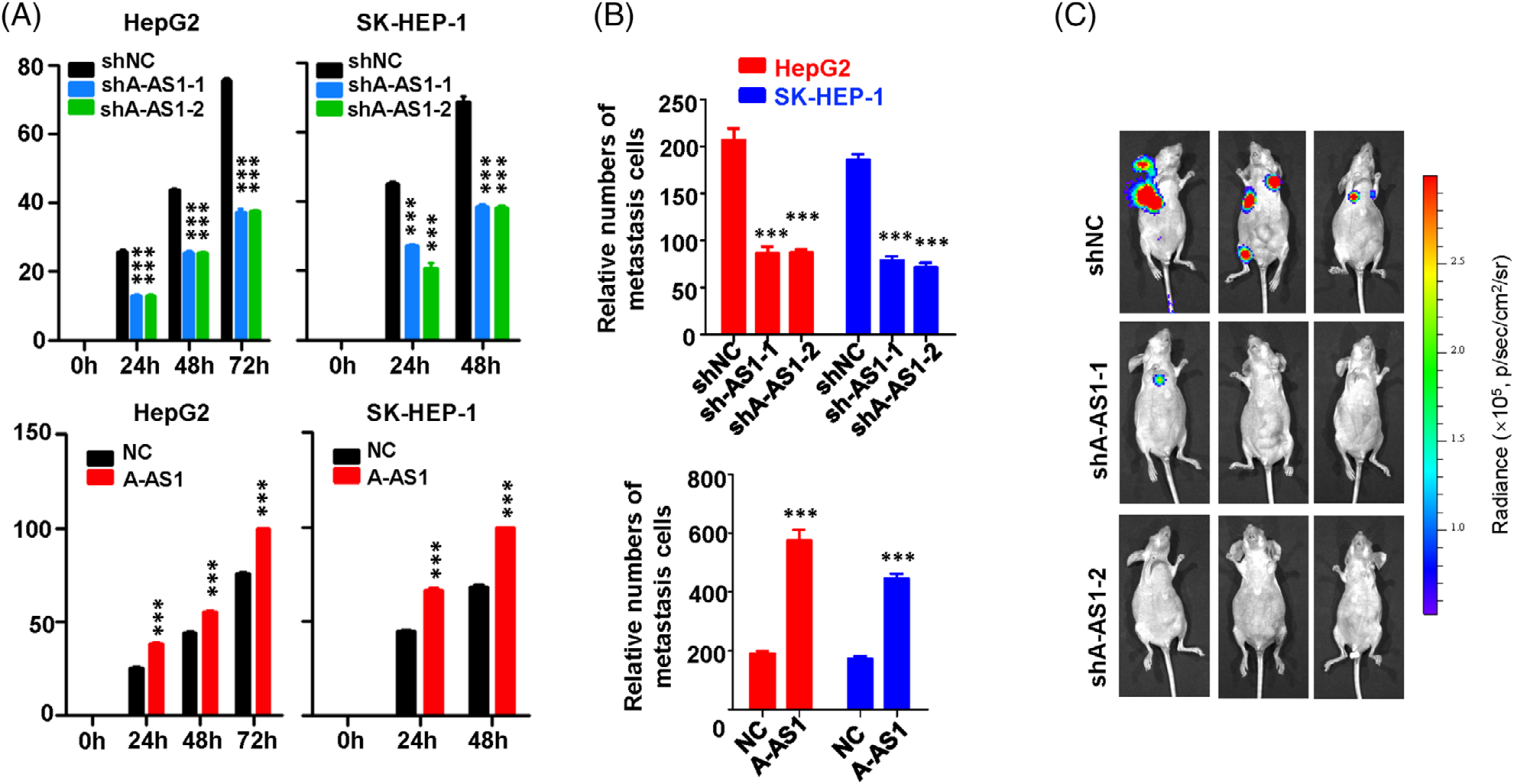
ARHGAP5‐AS1 reduces the migration, invasion, and metastasis capabilities of HCC cells. (A) In HepG2 and SK‐HEP‐1 cells, silencing of *ARHGAP5‐AS1* inhibited wound healing and the stably enforced ARHGAP5‐AS1 expression evidently accelerated wound healing. (B) ARHGAP5‐AS1 promoted the invasion abilities of HepG2 and SK‐HEP‐1 cells. (C) Fluorescent images of tumours in nude mice with tail vein injected SK‐HEP‐1 cells with or without knockdown of *ARHGAP5‐AS1*. Data information: Each value represents mean ± SD. The difference between the two groups was calculated using Student's *t*‐test. ***p < 0.001.

We then evaluated the effects of lncRNA ARHGAP5‐AS1 in metastatic behaviours of HCC cells ex vivo and in vivo. Interestingly, the stable silencing of ARHGAP5‐AS1 significantly impaired cell motility of HepG2 or SK‐HEP‐1 cells (both *p* < 0.001) (Figure 3A and Figure S2A). On the contrary, forced expression of ARHGAP5‐AS1 promoted migration of HepG2 or SK‐HEP‐1 cells (both *p* < 0.001) (Figure 3B and Figure S2B). The Matrigel invasion assays indicated that *ARHGAP5‐AS1*‐KD impaired the invasion of HCC cells (Figure 3B and Figure S2B). In contrast, overexpression of ARHGAP5‐AS1 accelerated the invasion of HCC cells (Figure 3B and Figure S2C,D). The in vivo HCC metastasis results revealed that silencing of ARHGAP5‐AS1 can significantly impair the distant metastasis of the lung and other organs of HCC cells after injection of malignant cells (Figure 3C). Collectively, these data demonstrated that lncRNA ARHGAP5‐AS1 could enhance motility and invasion of HCC cells ex vivo and in vivo.

### 3.4 ARHGAP5‐AS1 inhibited CSDE1 degradation via proteasome

**FIGURE 4:**
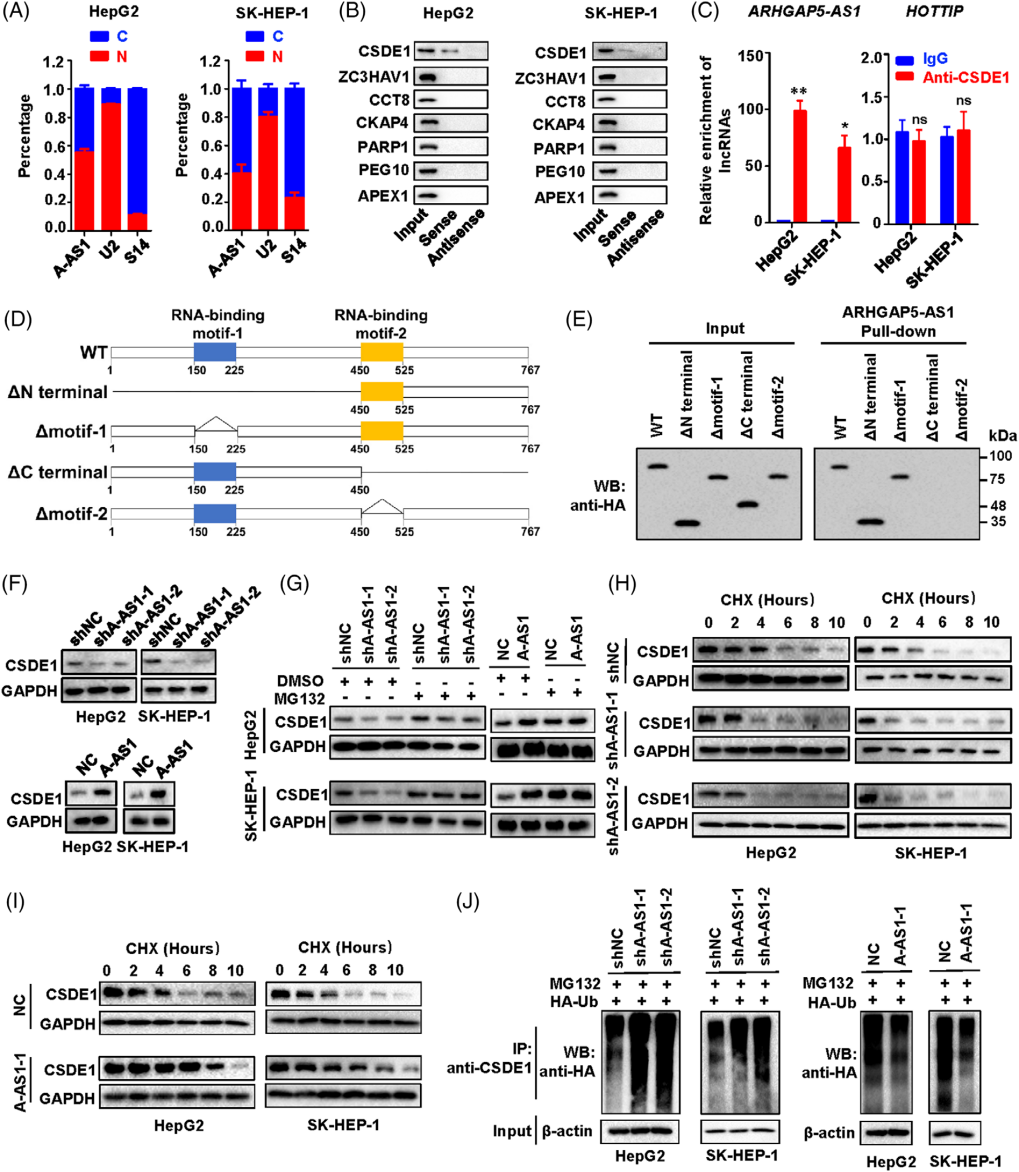
ARHGAP5‐AS1 interacts with CSDE1 and prevents CSDE1 degradation. (A) Cellular location of lncRNA ARHGAP5‐AS1 in HepG2 and SK‐HEP‐1 cells. S14 RNA or U2 RNA represents RNAs in the cytosolic fraction or the nuclear fraction. (B) ARHGAP5‐AS1 pull‐down followed by Western blot validated its interaction with CSDE1 and other candidate proteins identified by Mass spectrometry. (C) The RIP assays showed an association of CSDE1 with lncRNA ARHGAP5‐AS1 in HepG2 and SK‐HEP‐1 cells. Relative enrichment (means ± SD) represents RNA levels associated with CSDE1 relative to an input control from three independent experiments. IgG served as the control. LncRNA HOTTIP was used as the negative control. (D) Schematic diagram of HA‐tagged CSDE1 and its truncated forms used in ARHGAP5‐AS1 pull‐down assays. (E) Western blot analysis of HA‐tagged wild‐type (WT) CSDE1 and its truncated forms retrieved by in vitro transcribed biotinylated lncRNA ARHGAP5‐AS1. (F) Western blot analyses of CSDE1 protein in HCC cells with silenced expression of *ARHGAP5‐AS1* or the enforced expression of *ARHGAP5‐AS1*. (G) HepG2 and SK‐HEP‐1 cells after *ARHGAP5‐AS1*‐knockdown or stably overexpressing *ARHGAP5‐AS1* and control cells were treated with MG132 or vehicle. CSDE1 levels were measured by Western blot. (H,I) Western blot analyses of CSDE1 protein levels in HCC cells that stabilised either silenced *ARHGAP5‐AS1* or overexpressed *ARHGAP5‐AS1* treated with cycloheximide (CHX) for the indicated periods of time. (J) Western blot detection of the ubiquitination of CSDE1 protein in HepG2 and SK‐HEP‐1 cells with silenced expression of *ARHGAP5‐AS1* or the enforced expression of *ARHGAP5‐AS1* after transfection with HA‐Ubiquitin (HA‐Ub). Data information: The difference between the two groups was calculated using Student's *t*‐test. *p < 0.05, **p < 0.01.

Accumulating evidences indicated that lncRNAs could play their roles through interacting with various proteins during tumorigenesis.^27^, ^28^, ^29^ Therefore, we hypothesised that lncRNA ARHGAP5‐AS1 may act as scaffolds for binding certain protein(s) to promote HCC development. To verify this, we examined the cellular localization of ARHGAP5‐AS1 and found that ARHGAP5‐AS1 nearly equally exists in either the nucleus or the cytoplasm of HCC cells (Figure 4A). After examining the pulled‐down proteins by lncRNA ARHGAP5‐AS1 using mass spectrometry proteomics, we identified multiple cancer‐related proteins including CSDE1, ZC3HAV1, CCT8, CKAP4, PARP1, PEG10 and APEX1 in HepG2 (Table S6). Independent assays in HCC cells were performed and successfully validated CSDE1 among these candidate proteins (Figure 4B). In line with this, there was significant lncRNA ARHGAP5‐AS1 enrichment in the RNA‐CSDE1 complexes in HCC cell lines (both *p* < 0.01) (Figure 4C). During RIP, lncRNA HOTTIP was used as the negative control (Figure 4C). To explore the specific domains required for the interaction between lncRNA and CSDE1, we then constructed various truncated *CSDE1* (Figure 4D) and found that the CSDE1 RNA binding motif 2 (aa450‐525) is required for the interaction between lncRNA ARHGAP5‐AS1 and the protein (Figure 4E).

Intriguingly, silencing of ARHGAP5‐AS1 significantly suppressed CSDE1 protein levels in HCC cells (Figure 4F). Instead, the over‐expressed ARHGAP5‐AS1 markedly up‐regulated CSDE1 protein in HepG2 and SK‐HEP‐1 cells (Figure 4F). Treatment of the *ARHGAP5‐AS1*‐KD HCC cells with the 26S protostome inhibitor MG132 increased the expression of endogenous CSDE1 protein in comparison with the control HCC cells (Figure 4G). Conversely, MG132 abolished ARHGAP5‐AS1‐induced up‐regulation of CSDE1 protein in HCC cells (Figure 4G), elucidating that the lncRNA may regulate the proteasome degradation of CSDE1. To confirm this, we next detected CSDE1 expression in HepG2 and SK‐HEP‐1 cells treated with CHX, a protein synthesis inhibitor. The results of the Western Blot showed that the protein levels of CSDE1 declined much faster in the stable *ARHGAP5‐AS1*‐KD HCC cells than those in the control cells (Figure 4H). In contrast, treatment of HCC cells overexpressing ARHGAP5‐AS1 with CHX led to an obviously longer half‐life of CSDE1 protein than in control cells (Figure 4I). We then investigated whether lncRNA ARHGAP5‐AS1‐controlled degradation of CSDE1 was mediated by ubiquitination of CSDE1. After endogenous CSDE1 was immunoprecipitated in HA‐Ub‐transfected HepG2 or SK‐HEP‐1 cells, evidently increased ubiquitination levels of CSDE1 protein were observed in the stable *ARHGAP5‐AS1*‐KD HCC compared to controls (Figure 4J). In line with this, the ubiquitination of CSDE1 was decreased in cells overexpressing ARHGAP5‐AS1 compared to controls (Figure 4J). Taken together, these results elucidated that lncRNA ARHGAP5‐AS1 stabilise CSDE1 protein by inhibiting its proteasome degradation.

### 3.5 ARHGAP5‐AS1 interrupted interactions of CSDE1 with its E3 ligase TRIM28

**FIGURE 5:**
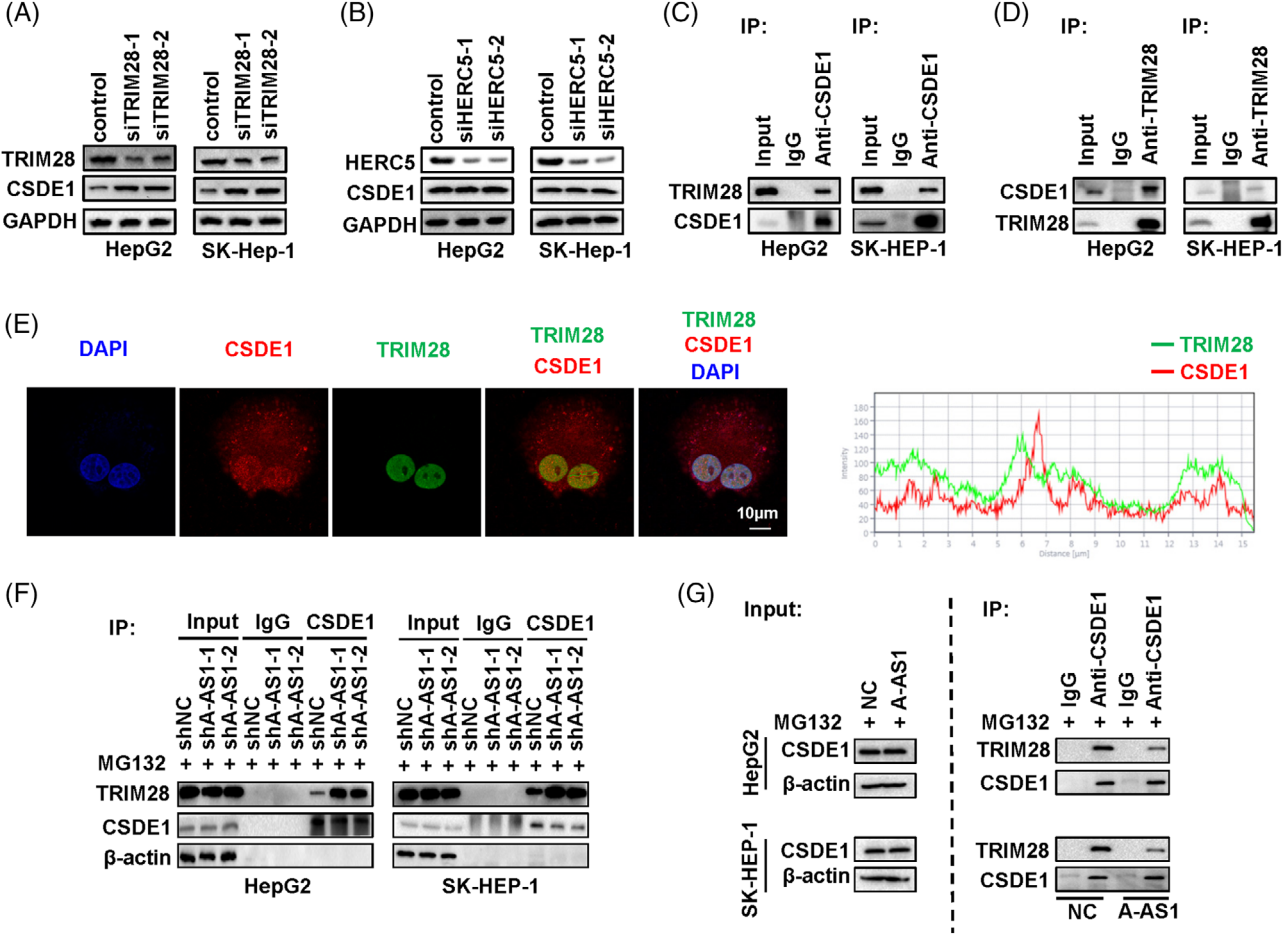
ARHGAP5‐AS1 interrupts the binding of CSDE1 with its E3 ligase TRIM28. (A) Knocking‐down of *TRIM28* expression obviously elevated CSDE1 protein levels in HCC cells in comparison with the control cells. (B) Silenced expression of *HERC5* did not impact CSDE1 protein levels in HCC cells. (C,D) Interactions between CSDE1 and TRIM28 were verified via Co‐IP assays in HepG2 and SK‐HEP‐1 cells. (E) The immunofluorescence assays showed co‐localization of TRIM28 and CSDE1 proteins in HCC cells. We used ZEISS Zen 3.3 (blue edition) to generate the line profiles of fluorescent intensities in confocal images. (F,G) LncRNA ARHGAP5‐AS1 considerably attenuated interactions between CSDE1 and TRIM28 in HCC cells.

To disclose how ARHGAP5‐AS1 retards the proteasome degradation of CSDE1, we systematically evaluated proteins precipitated by CSDE1 in HepG2 cells through mass spectrometry. Among all proteins identified, there were only two E3 ligases (TRIM28 and HERC5) (Table S7). To confirm if TRIM28 or HERC5 is the E3 ligase of CSDE1, we firstly examined CSDE1 levels in HCC cells with silenced expression of TRIM28 or HERC5 (Figure 5A,B). After the knocking‐down of TRIM28 expression, elevated CSDE1 protein levels could be detected in HCC cells in comparison with the control cells (Figure 5A). However, no such expression changes were observed after silencing of HERC5 expression in HCC cells (Figure 5B). Importantly, endogenous TRIM28 can be immunoprecipitated with CSDE1 in HepG2 or SK‐HEP‐1 cells (Figure 5C). Endogenous CSDE1 could also be precipitated with TRIM28 in HepG2 or SK‐HEP‐1 cells (Figure 5D). Immunofluorescence assays revealed that TRIM28 and CSDE1 exhibited evident co‐localization in HCC cells (Figure 5E). Similarly, RNA FISH assays indicated the co‐localization of lncRNA ARHGAP5‐AS1 and CSDE1 protein in cells (Figure S3). These data indicated that TRIM28 might be the potential E3 ligase of CSDE1 in HCC. We next investigated if lncRNA ARHGAP5‐AS1 influences the binding of CSDE1 with TRIM28 in HCC cells. More TRIM28 protein could be precipitated with CSDE1 in the stable *ARHGAP5‐AS1*‐KD HepG2 or SK‐HEP‐1 cells compared to controls (Figure 5F). Conversely, there was less TRIM28 protein precipitated with CSDE1 in the stably *ARHGAP5‐AS*1‐OE HCC cells in comparison with the control cells (Figure 5G). Taking together, these findings suggested that lncRNA ARHGAP5‐AS1 promotes CSDE1 stabilization by attenuating its interactions with the E3 ligase TRIM28.

### 3.6 The ARHGAP5‐AS1‐CSDE1 axis promoted protein expression of VIM and RAC1 as well as phosphorylation of ERK in HCC cells

**FIGURE 6:**
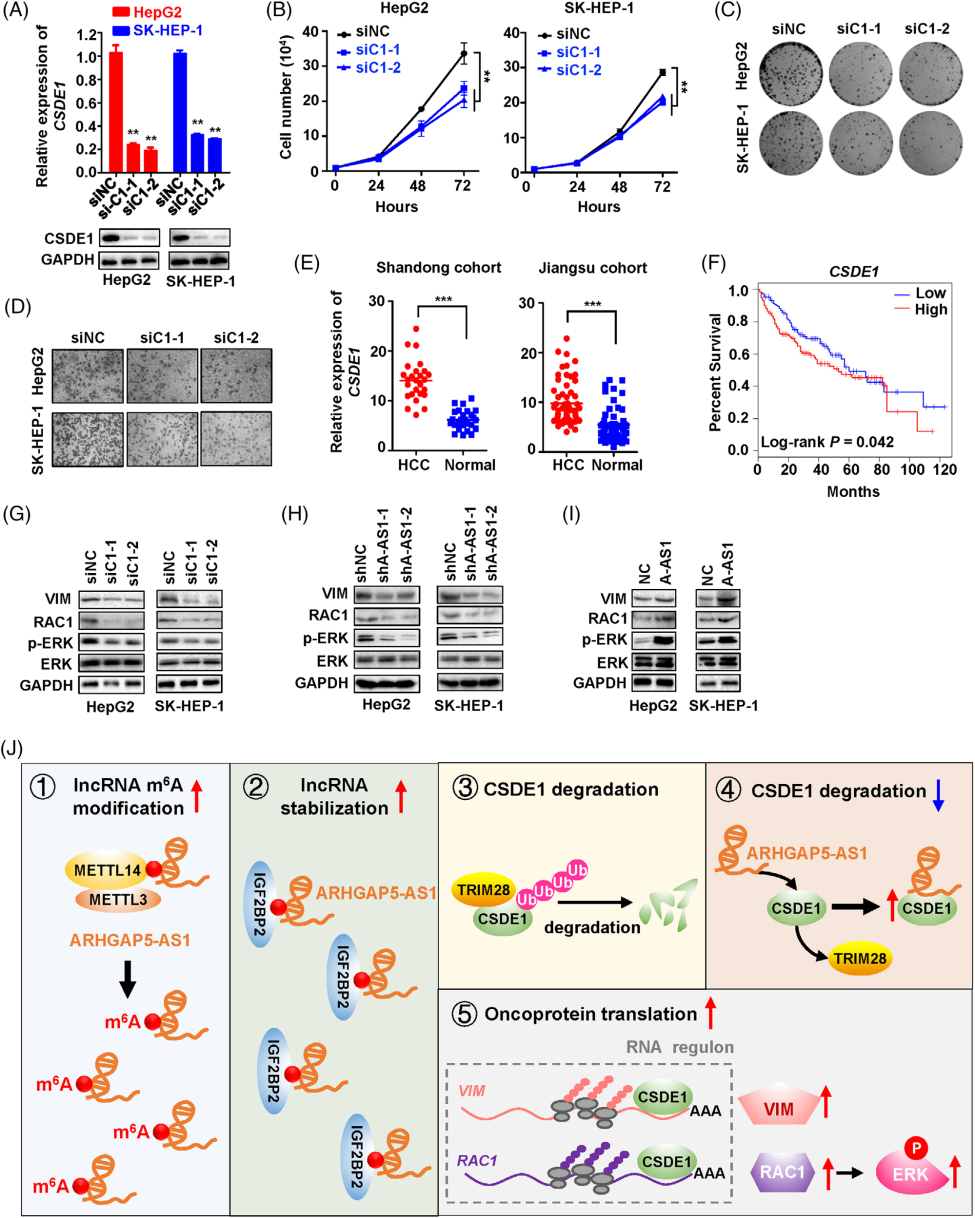
ARHGAP5‐AS1‐induced upregulation of CSDE1 promotes translation of *VIM* and *RAC1* as well as stimulation of the ERK signalling. (A,B) Knockdown of *CSDE1* with its siRNAs (siC1‐1 and siC1‐2) substantially suppressed the proliferation of HCC cells. (C) Effects of *CSDE1*‐knockdown on the colony formation of HepG2 and SK‐HEP‐1 cells. (D) Silencing of *CSDE1* reduced the invasion abilities of HCC cells. (E) Expression of *CSDE1* was compared between normal and cancer samples in the Shandong cohort and Jiangsu cohort. (F) The Kaplan–Meier analyses of the survival time of TCGA HCC patients were stratified according to *CSDE1* expression levels. (G) Silencing of *CSDE1* evidently decreased levels of VIM and RAC1 as well as phosphorylated ERK1/2 (Thr202/Tyr204) in HCC cells. (H) HepG2 or SK‐HEP‐1 cells with stable *ARHGAP5‐AS1*‐knockdown showed markedly downregulated expression of VIM and RAC1 as well as phosphorylated ERK1/2. (I) Ectopic *ARHGAP5‐AS1* expression obviously increased levels of VIM and RAC1 as well as phosphorylated ERK1/2. (J) Graphical representation of the functions of lncRNA ARHGAP5‐AS1 in HCC. (i) METTL14 is the m^6^A writer of lncRNA ARHGAP5‐AS1. (ii) IGF2BP2 acts as the m^6^A reader to stabilise ARHGAP5‐AS1 in HCC. (iii) The E3 ligase TRIM28 promotes ubiquitination and degradation of oncoprotein CSDE1 via the ubiquitin‐proteasome pathway. (iv) ARHGAP5‐AS1 attenuates the binding of CSDE1 with TRIM28. v) ARHGAP5‐AS1‐induced upregulation of CSDE1 promotes translation of *VIM* and *RAC1* as well as stimulation of the ERK signalling. Data information: The difference between the two groups was calculated using Student's *t*‐test. **p < 0.01, ***p < 0.001. Data shows one representative of three independent experiments with three biological replicates.

Multiple lines of evidence demonstrated that the RNA‐binding protein CSDE1 acts as an oncogene in cancers and regulates the translation and stability of mRNAs at the post‐transcriptional level.^30^, ^31^, ^32^ Indeed, silencing of *CSDE1* profoundly suppressed proliferation and clonogenicity of HCC cells (Figure 6A–C and Figure S4A). Moreover, the transwell assays indicated that siRNAs of *CSDE1* could markedly inhibit the invasion capability of HCC cells (Figure 6D and Figure S4B). In line with these data, remarkably elevated *CSDE1* expression in HCC tissues was detected in comparison with the normal specimens in both cohorts (*p* < 0.001). Aberrantly high expression of *CSDE1* in the TCGA LIHC cohort was associated with evidently shortened OS of patients (Figure 6F), indicating the oncogenic nature of *CSDE1* in HCC. Rescue assays indicated that silencing of *CSDE1* with siRNAs significantly inhibited the proliferation of HCC cells with stably overexpressed *ARHGAP5‐AS1* (both *p* < 0.05) (Figure S5).

It has been found that the regulation of *VIM* and *RAC1* mRNA translation by CSDE1 contributes to melanoma metastasis.^30^ As shown in Figure 6G, we observed evidently decreased protein levels of VIM (Vimentin) and RAC1 in HCC cells after silencing of *CSDE1*. Likewise, there is a downregulated expression of VIM and RAC1 in the stable *ARHGAP5‐AS1*‐KD HepG2 or SK‐HEP‐1 cells compared to controls (Figure 6H); whereas, ectopic ARHGAP5‐AS1 expression markedly elevated expression of VIM and RAC1 (Figure 6I). Interestingly, the knocking‐down of ARHGAP5‐AS1 or CSDE1 reduced the phosphorylation of ERK1/2 (Thr202/Tyr204) in cells (Figure 6G,H). Ectopic ARHGAP5‐AS1 obviously enhanced ERK1/2 phosphorylation in cells (Figure 6I). Together, these data demonstrated the key part of lncRNA ARHGAP5‐AS1 in stabilizing CSDE1 protein, promoting the translation of *VIM* and *RAC1* as well as activating the ERK signalling in HCC (Figure 6J).

## 4 DISCUSSION

After the genome‐wide screening of lncRNAs in HCC via m^6^A‐seq and RNA‐seq, we successfully identified ARHGAP5‐AS1 as a novel m^6^A‐modified lncRNA. METTL14 is the m^6^A writer of ARHGAP5‐AS1 and IGF2BP2 acts as its m^6^A reader to stabilise the lncRNA. Increased ARHGAP5‐AS1 expression was detected in cancerous specimens and associated with evidently shortened survival time of HCC patients. Consistently, lncRNA ARHGAP5‐AS1 exhibited strong oncogenic potentials ex vivo and in vivo. In particular, ARHGAP5‐AS1 could interrupt interactions between CSDE1 and TRIM28, stabilise oncoprotein CSDE1, boost translation of *VIM* and *RAC1* mRNAs, stimulate the ERK signalling and, thus, accelerate HCC progression.

It is becoming more and more clear that the expression of certain lncRNAs is precisely regulated by their m^6^A modification levels during hepatocarcinogenesis.^33^, ^34^, ^35^, ^36^, ^37^ For example, METTL3‐mediated m^6^A modification of LINC00958 led to increased gene expression through stabilizing the lncRNA.^33^ Oncogenic LINC00958 is a lipogenesis‐related RNA which can sponge miR‐3619‐5p to elevate hepatoma‐derived growth factor (HDGF) expression and accelerated HCC development.^33^ Similarly, Jia et al. found that elevated expression of lncRNA LNCAROD was maintained by its increased m^6^A methylation.^36^ Enhancement of glycolysis, which is mediated by pyruvate kinase isoform M2 (PKM2), is vital for tumourigenesis. LNCAROD interacts with SRSF3 to induce switching from PKM to PKM2 and preserves expression levels of PKM2 by sponging miR‐145‐5p in HCC. As a result, LNCAROD could promote proliferation, invasion, and chemoresistance of HCC cells.^33^ However, there is still no systematical screening of m^6^A‐methylated lncRNAs in HCC cells up until now. After genome‐wide analyses of HCC m^6^A‐seq and RNA‐seq data, we successfully identified ARHGAP5‐AS1 as a novel m^6^A‐methylated lncRNA. Silencing of METTL14 results in evidently downregulated m^6^A and expression levels of ARHGAP5‐AS1. IGF2BP2, the m^6^A reader of ARHGAP5‐AS1, sustains its high levels in HCC.

As the antisense non‐coding transcript of *ARHGAP5*, lncRNA ARHGAP5‐AS1 has been revealed to contribute to the development of breast cancer and gastric cancer.^38^, ^39^ After analysing the RNA‐seq data of breast cancer MDA‐MB‐231 cells and the highly metastatic derivative MDA‐MB‐231‐LM2 cells (LM2), Wang et al. found that ARHGAP5‐AS1 expression level was markedly reduced in LM2 cells.^39^ It has been reported that ARHGAP5‐AS1 could suppress cell migration through downregulating SMAD7 expression in breast cancer cells.^39^ On the contrary, ARHGAP5‐AS1 is upregulated in chemoresistant gastric cancer cells and the knocking down of ARHGAP5‐AS1 can effectively reverse chemoresistance.^38^ High ARHGAP5‐AS1 expression was significantly associated with the poor prognosis of patients who suffered from gastric cancer.^38^ Consistently, we also observed that lncRNA ARHGAP5‐AS1 acts as an oncogenic modulator of HCC progression.

Overexpressed CSDE1 protein has been suggested as a vital component during tumourigenesis including melanoma and glioma.^30^, ^31^, ^32^ CSDE1 is an RNA‐binding protein and coordinates oncogenic RNA regulons, such as *VIM* and *RAC1* genes. Through enhancing translation elongation of *VIM* and *RAC1* mRNAs, CSDE1 upregulates the expression of VIM and RAC1, and, thus, promotes HCC development.^30^, ^32^ In line with these findings, we found that CSDE1 also plays its role as an oncogene in HCC and lncRNA ARHGAP5‐AS1 could interrupt the binding of CSDE1 with its E3 ligase TRIM28, stabilise CSDE1 protein, elevate expression of VIM and RAC1, and stimulate ERK signalling. This may underline mechanisms of how ARHGAP5‐AS1 contributes to HCC proliferation and metastasis ex vivo and in vivo.

## 5 CONCLUSIONS

In conclusion, we identified an oncogenic lncRNA, ARHGAP5‐AS1, by comprehensively analysing m^6^A‐modified RNAs and profiling lncRNA expression in HCC cells. Significantly elevated expression of lncRNA ARHGAP5‐AS1 due to its high m^6^A methylation levels accelerates the translation of *VIM* and *RAC1* and stimulation of the ERK signalling pathway, which promotes cell proliferation and metastasis. Our current study reveals a novel paradigm in m^6^A‐modified lncRNAs controlling CSDE1‐mediated oncogenic RNA regulons and highlights lncRNAs as potential targets for future therapeutics against HCC.

## CONFLICT OF INTEREST

The authors declare no competing financial interests.

## Supporting information

Supporting Information

Supporting Information

Supporting Information

Supporting Information

Supporting Information

Supporting Information

Supporting Information
