# Supplementary material for: N6‐methyladenosine‐modified lncRNA ARHGAP5‐AS1 stabilises CSDE1 and coordinates oncogenic RNA regulons in hepatocellular carcinoma
Source: Clin Transl Med. 2022 Nov 10;12(11):e1107. doi: 10.1002/ctm2.1107 (PMC9647857; doi:10.1002/ctm2.1107)
Supplement: Supplementary file 1 — Supporting Information [file CTM2-12-e1107-s003.docx]

**Supplementary Table 1.** Antibodies used in the study

| Name | Company | Catalog Number |
| --- | --- | --- |
| Anti-N6-methyladenosine | Abcam | ab190886 |
| IgG | Invitrogen | 02-6102 |
| METTL14 | Proteintech | 26158-1-AP |
| IGF2BP1 | Proteintech | 22803-1-AP |
| IGF2BP2 | Proteintech | 11601-1-AP |
| IGF2BP3 | Proteintech | 14642-1-AP |
| GAPDH | Proteintech | 10494-1-AP |
| CSDE1 | Abcam | ab201688 |
| ZC3HAV1 | Proteintech | 16820-1-AP |
| CCT8 | Proteintech | 12263-1-AP |
| CKAP4 | Proteintech | 16686-1-AP |
| PARP1 | Proteintech | 13371-1-AP |
| PEG10 | Proteintech | 14412-1-AP |
| APEX1 | Proteintech | 10203-1-AP |
| Anti-HA | Sigma | H6908 |
| β-actin | Proteintech | 20536-1-Ap |
| TRIM28 (Mouse) | Proteintech | 66630-1-Ig |
| TRIM28 (Rabbit) | Abcam | ab109545 |
| HERC5 | Proteintech | 22692-1-AP |
| USP10 | Abcam | ab109219 |
| Vimentin | CST | 5741 |
| RAC1 | Proteintech | 24072-1-AP |
| p-ERK | CST | 4370S |
| ERK | CST | 4695S |
| CoraLite488-conjugated Affinipure Goat Anti-Mouse IgG(H+L) | Proteintech | SA00013-1 |
| CoraLite488-conjugated Goat Anti-Rabbit IgG(H+L) | Proteintech | SA00013-2 |
| CoraLite594-conjugated Goat Anti-Rabbit IgG(H+L) | Proteintech | SA00013-4 |

**Supplementary Table 2.** Primers for RT-qPCR

| Name | Primer Sequence |
| --- | --- |
| ARHGAP5-AS1-F | TCGCTCGCCAACTACAGA |
| ARHGAP5-AS1-R | TCGCTCCACTTTCACAGAAC |
| LINC00152-F | ACTGGGAGATGAAACAGGAAGC |
| LINC00152-R | GGCAGACCACCCGCAAAT |
| C1QTNF1-AS1-F | GCTTGGGACAGGTGGAGA |
| C1QTNF1-AS1-R | ACAATCAGGCTTTCATTGGAT |
| LINC00969-F | AGCAGATCCGTGGTTCCC |
| LINC00969-R | CCGTCCCAAGACAGCAAAGG |
| USP27X-AS1-F | AAAGGAGGATGCGTGGTTC |
| USP27X-AS1-R | CAGTCTGGAGGTGGGATGAG |
| NDUFB2-AS1-F | GGGCTTGACTCCATTCGC |
| NDUFB2-AS1-R | CCAACCACGGGCTTTCTTC |
| TEN1-CDK3-F | TTGCCCGGTGCCTTCTGT |
| TEN1-CDK3-R | GCTGCCCTGTCTCCCTGTTC |
| ABALON-F | TGTGGGTCTTACGAAGGTCTG |
| ABALON-R | GGCGGATTTGAATGTAGGTG |
| ARHGAP5-AS1-mutF | CCAGAAGTACATTTACGG |
| ARHGAP5-AS1-mutR | AGTGCAGGGTCCGAGGTATT |
| METTL14-F | GAGTGTGTTTACGAAAATGGGGT |
| METTL14-R | CCGTCTGTGCTACGCTTCA |
| IGF2BP2-F | AGCTAAGCGGGCATCAGTTTG |
| IGF2BP2-R | CCGCAGCGGGAAATCAATCT |
| S14-F | GGCAGACCGAGATGAATCCTC |
| S14-R | CAGGTCCAGGGGTCTTGGTCC |
| U2-F | CATCGCTTCTCGGCCTTTTG |
| U2-R | TGGAGGTACTGCAATACCAGG |
| GAPDH-F | ATTCTCTGATTTGGTCGTATTGGG |
| GAPDH-R | ATGACAAGCTTCCCGTTCTC |
| HOTTIP-F | CCTAAAGCCACGCTTCTTTG |
| HOTTIP-R | TGCAGGCTGGAGATCCTACT |
| CSDE1-F | AAACCAGAATGACCCATTGC |
| CSDE1-R | ATTTGTCACGTCGGTCTGTTG |

**Supplementary Table 3.** Sequences of siRNAs and shRNAs

| Name | siRNA or shRNA sequence |
| --- | --- |
| NC | Sense 5'-UUCUCCGAACGUGUCACGUTT-3' |
|  | Antisense 5'-ACGUGACACGUUCGGAGAATT-3' |
| siMETTL14-1 | Sense 5'-CCUCCUCCCAAAUCUAAAUTT-3' |
| (siM14-1) | Antisense 5'-AUUUAGAUUUGGGAGGAGGTT-3' |
| siMETTL14-2 | Sense 5'-GGCUAAAGGAUGAGUUAAUTT-3' |
| (siM14-2) | Antisense 5'-AUUAACUCAUCCUUUAGCCTT-3' |
| siIGF2BP2-1 | Sense 5'-GCCGCAUGAUUCUUGAAAUTT-3' |
|  | Antisense 5'-AUUUCAAGAAUCAUGCGGCTT-3' |
| siIGF2BP2-2 | Sense 5'-CCCGCAUCAUCACUCUUAUTT-3' |
|  | Antisense 5'-AUAAGAGUGAUGAUGCGGGTT-3' |
| siCSDE1-1 | Sense 5'-CCACAACAAUGGACAUAAUTT-3' |
| (siC1-1) | Antisense 5'-AUUAUGUCCAUUGUUGUGGTT-3' |
| siCSDE1-2 | Sense 5'-CCGAGAAAUGGGUGUGAUUTT-3' |
| (siC1-2) | Antisense 5'-AAUCACACCCAUUUCUCGGTT-3' |
| shNC | 5'-CCGGTAGTCGCATACGGAACATTCGCTCGAGCGAATGT  TCCGTATGCGACTATTTTTT-3' |
| shA-AS1-1 | 5'-GCCACTACCAGCCTAACTTTACTCGAGTAAAGTTAGG  CTGGTAGTGGCTTTTTT-3' |
| shA-AS1-2 | 5'-GCTTCCCTCTTTCTCCGTATTCTCGAGAATACGGAGA  AAGAGGGAAGCTTTTTT-3' |
| siTRIM28-1 | Sense 5'-CACUAGCUGUGAGGAUAAUTT-3' |
|  | Antisense 5'-AUUAUCCUCACAGCUAGUGTT-3' |
| siTRIM28-2 | Sense 5'-CUGAGGACUACAACCUUAUTT-3' |
|  | Antisense 5'-AUAAGGUUGUAGUCCUCAGTT-3' |
| siHERC5-1 | Sense 5'-GACGCCGAAAUGCAUUAAATT-3' |
|  | Antisense 5'-UUUAAUGCAUUUCGGCGUCTT-3' |
| siHERC5-2 | Sense 5'-GGGACAGAAACGACACAAATT-3' |
|  | Antisense 5'-UUUGUGUCGUUUCUGUCCCTT-3' |

**Supplementary Table 4.** Associations between candidate lncRNAs and prognosis of LIHC patients in TCGA

| LncRNAs | Overall Survival | | |  | Disease Free Survival | | |
| --- | --- | --- | --- | --- | --- | --- | --- |
|  | HR | *P*(HR) | Logrank *P* |  | HR | *P*(HR) | Logrank *P* |
| ARHGAP5-AS1 | 1.6 | 0.0059 | 0.0053 |  | 1.7 | 0.00065 | 0.00059 |
| C1QTNF1-AS1 | 1.2 | 0.39 | 0.38 |  | 0.73 | 0.043 | 0.042 |
| COX10-AS1 | 0.86 | 0.39 | 0.38 |  | 0.96 | 0.78 | 0.77 |
| DARS-AS1 | 1.3 | 0.21 | 0.2 |  | 1.1 | 0.56 | 0.56 |
| DHRS4-AS1 | 0.92 | 0.65 | 0.65 |  | 0.95 | 0.74 | 0.74 |
| ENTPD1-AS1 | 1.3 | 0.19 | 0.19 |  | 1.2 | 0.19 | 0.19 |
| FAM157HG | \ | \ | \ |  | \ | \ | \ |
| LINC00152 | 2 | 0.00019 | 0.00014 |  | 1.4 | 0.047 | 0.047 |
| LINC00969 | 1.3 | 0.16 | 0.16 |  | 1.4 | 0.018 | 0.018 |
| LINC01146 | 0.73 | 0.082 | 0.08 |  | 0.82 | 0.19 | 0.19 |
| MIR22HG | 0.82 | 0.25 | 0.25 |  | 0.75 | 0.057 | 0.057 |
| MTR163AHG | \ | \ | \ |  | \ | \ | \ |
| MZF1-AS1 | 1.2 | 0.32 | 0.32 |  | 1.1 | 0.42 | 0.41 |
| NDUFB2-AS1 | 1.7 | 0.0035 | 0.003 |  | 1.4 | 0.041 | 0.041 |
| SLCO4A1-AS1 | \ | \ | \ |  | \ | \ | \ |
| STX16-NPEPL1 | 0.89 | 0.53 | 0.53 |  | 0.99 | 0.92 | 0.93 |
| TEN1-CDK3 | 1.2 | 0.38 | 0.38 |  | 1.5 | 0.011 | 0.011 |
| THAP9-AS1 | 1.2 | 0.45 | 0.45 |  | 1.2 | 0.18 | 0.18 |
| TSPEAR-AS1 | 0.71 | 0.054 | 0.052 |  | 1 | 0.95 | 0.95 |
| TUG1 | 1.3 | 0.11 | 0.11 |  | 1.2 | 0.18 | 0.18 |
| USP27X-AS1 | 1.5 | 0.027 | 0.026 |  | 0.97 | 0.84 | 0.84 |
| ABALON | 1.5 | 0.037 | 0.036 |  | 1.1 | 0.46 | 0.46 |

**Supplementary Table 5.** The relevant clinic-pathological characteristics of HCC cases stratified by the ARHGAP5-AS1 expression

| **Patient characteristics** | **Number** | **ARHGAP5-AS1 expression** | | ***P* value** |
| --- | --- | --- | --- | --- |
|  |  | **Low (%)** | **High (%)** |  |
| Age (years) |  |  |  |  |
| ≤57 | 43 | 20(46.5) | 23(53.5) | 0.748 |
| >57 | 42 | 21(50.0) | 21(50.0) |  |
| Sex |  |  |  |  |
| Female | 13 | 6(46.2) | 7(53.8) | 0.728 |
| Male | 72 | 37(51.4) | 35(48.6) |  |
| HBsAg |  |  |  |  |
| Negative | 8 | 4(50.0) | 4(50.0) | n.c. |
| Positive | 77 | 38(49.4) | 39(50.6) |  |
| Cirrhosis |  |  |  |  |
| No | 14 | 6(42.9) | 8(57.1) | 0.592 |
| Yes | 71 | 36(50.7) | 35(49.3) |  |
| ALT (U/L) |  |  |  |  |
| ≤75 | 68 | 32(47.1) | 36(52.9) | 0.664 |
| >75 | 17 | 9(52.9) | 8(47.1) |  |
| AFP (ng/mL) |  |  |  |  |
| ≤20 | 25 | 13(52.0) | 12(48.0) | 0.758 |
| >20 | 60 | 29(48.3) | 31(51.7) |  |
| Tumor size (cm) |  |  |  |  |
| ≤5 | 43 | 22(51.2) | 21(48.8) | 0.585 |
| >5 | 42 | 19(45.2) | 23(54.8) |  |
| Tumor number |  |  |  |  |
| Single | 66 | 34(51.5) | 32(48.5) | 0.750 |
| Multiple | 19 | 9(47.4) | 10(52.6) |  |
| TNM stage |  |  |  |  |
| I/II | 66 | 42(63.6) | 24(36.4) | 0.013 |
| III/IV | 19 | 6(31.6) | 13(68.4) |  |

HBsAg, hepatitis B surface antigen; n.c., not calculated; AFP, alpha-fetoprotein; ALT, alanine aminotransferase; TNM, tumor-node-metastasis

**Supplementary Table 6.** Mass spectrometry of proteins pulled-down by lncRNA ARHAGP5-AS1 in HepG2 cells

| **No.** | **Gene names** | **Unique peptides** | **LFQ intensity Sense** | **LFQ intensity Antisense** |
| --- | --- | --- | --- | --- |
| **1** | **ZC3HAV1** | **5** | **61946000** | **0** |
| **2** | **CCT8** | **5** | **39252000** | **0** |
| 3 | DNAPTP6 | 4 | 60602000 | 0 |
| **4** | **CKAP4** | **4** | **35505000** | **0** |
| 5 | UBTF | 4 | 29520000 | 0 |
| **6** | **PARP1** | **3** | **11847000** | **0** |
| **7** | **PEG10** | **3** | **44119000** | **0** |
| 8 | ZFR | 3 | 11340000 | 0 |
| **9** | **APEX1** | **3** | **26155000** | **0** |
| 10 | TUBB2C | 3 | 65385000 | 0 |
| 11 | SLIRP | 3 | 66085000 | 0 |
| 12 | RTCB | 3 | 72014000 | 0 |
| **13** | **CSDE1** | **2** | **5500000** | **0** |
| 14 | RBM14 | 2 | 5060900 | 0 |
| 15 | HIST1H1E | 2 | 37321000 | 0 |

**Supplementary Table 7.** Mass spectrometry of two E3 ubiquitin ligase proteins immunoprecipitated by CSDE1 in HepG2 cells

| **No.** | **Gene names** | **Unique peptides** | **LFQ intensity CSDE1** | **LFQ intensity IgG** |
| --- | --- | --- | --- | --- |
| 1 | TRIM28 | 2 | 30086000 | 0 |
| 2 | HERC5 | 2 | 7468600 | 0 |
