# Supplementary figures and images for: N6‐methyladenosine‐modified lncRNA ARHGAP5‐AS1 stabilises CSDE1 and coordinates oncogenic RNA regulons in hepatocellular carcinoma
Source: Clin Transl Med. 2022 Nov 10;12(11):e1107. doi: 10.1002/ctm2.1107 (PMC9647857; doi:10.1002/ctm2.1107)

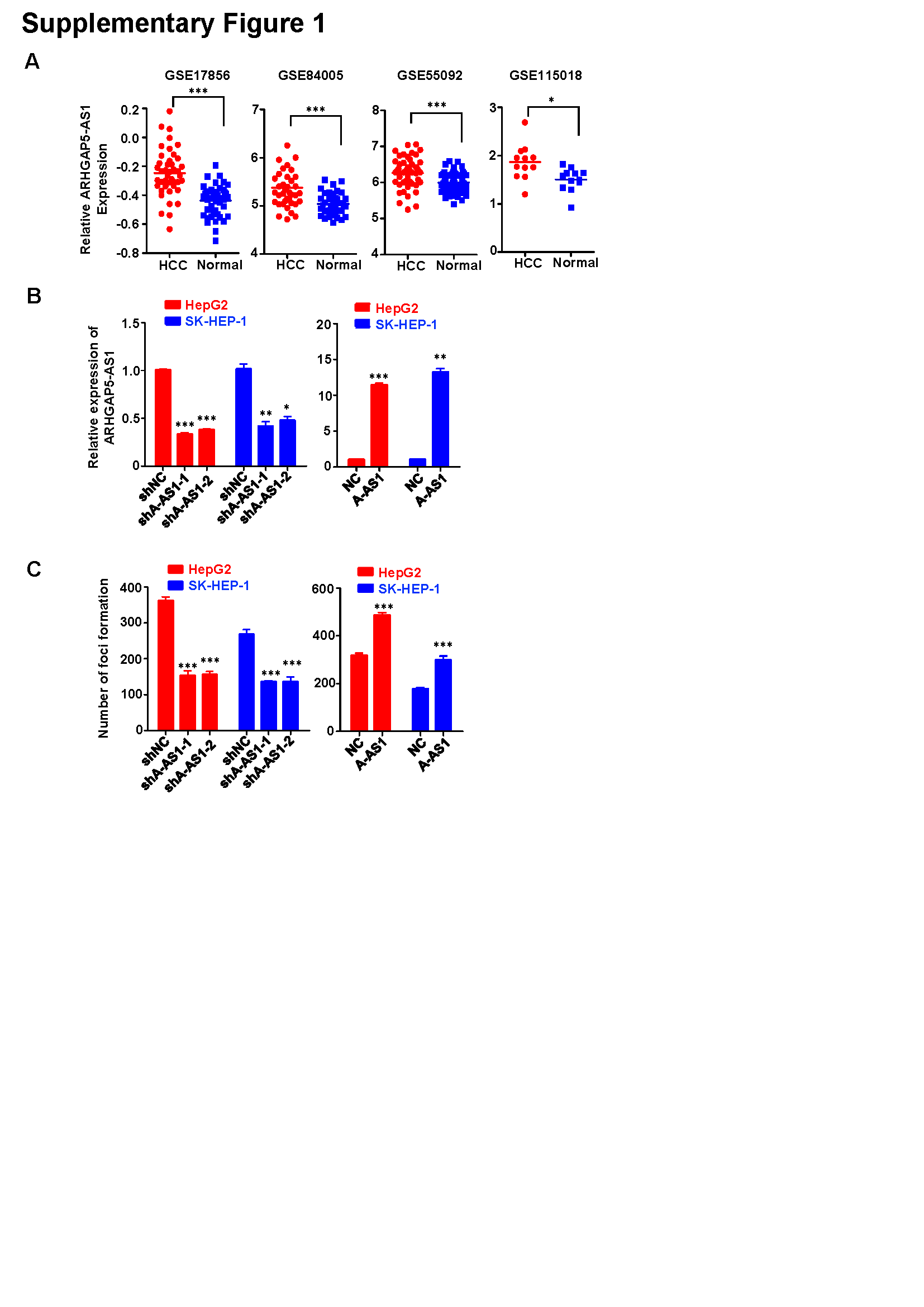

Supplement: Supplementary file 2 — Supporting Information [file CTM2-12-e1107-s002.tiff]

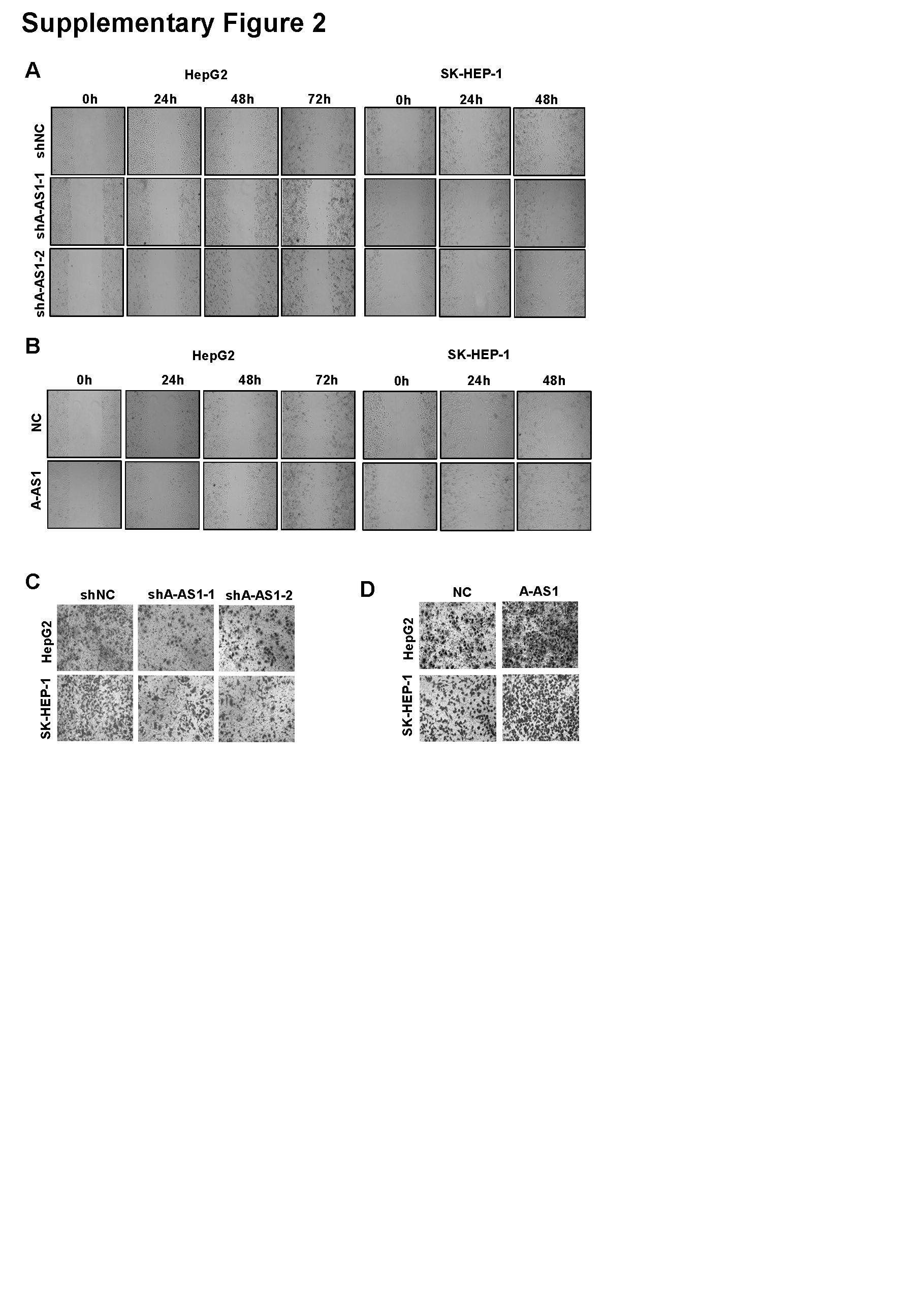

Supplement: Supplementary file 3 — Supporting Information [file CTM2-12-e1107-s001.tiff]

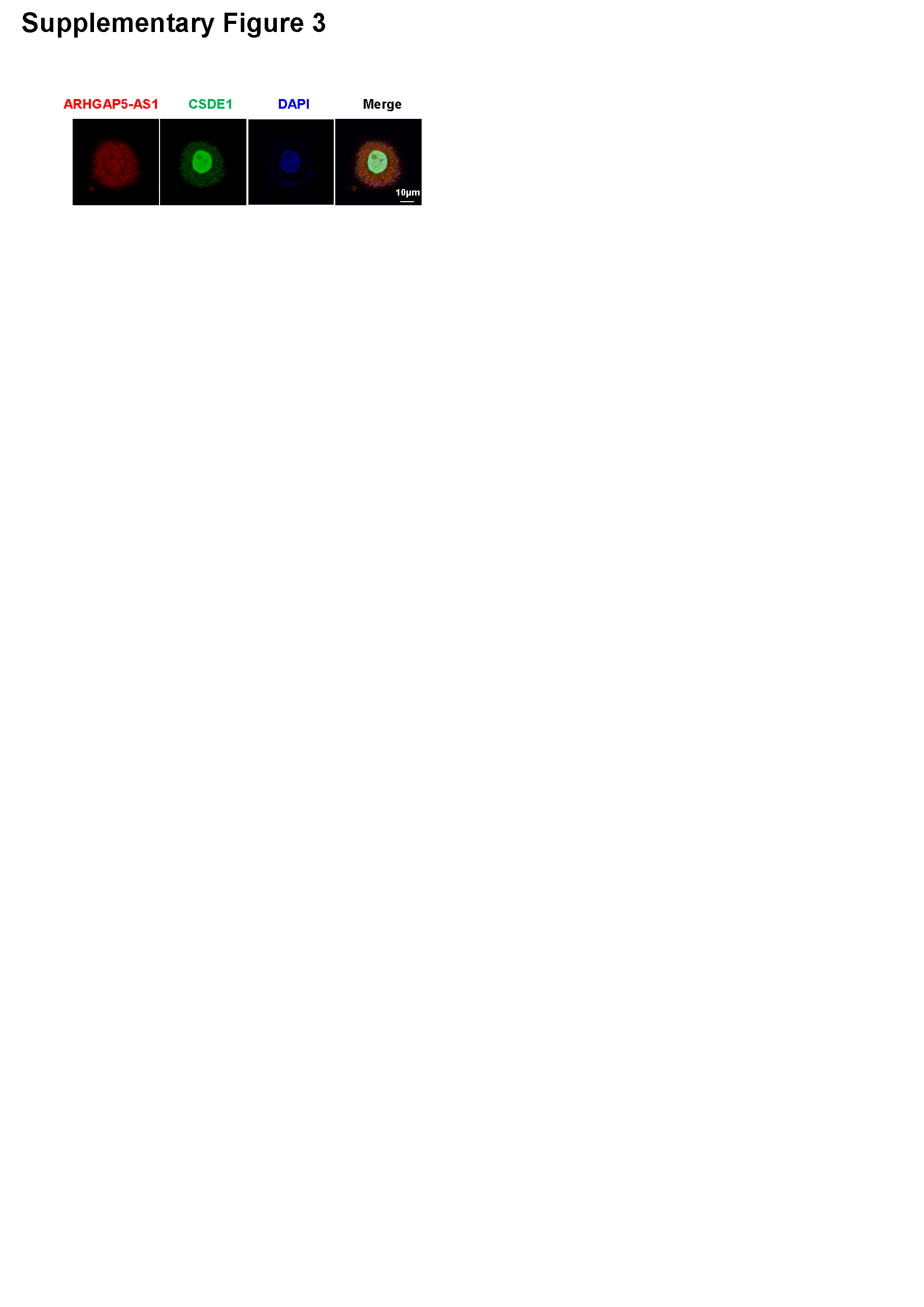

Supplement: Supplementary file 4 — Supporting Information [file CTM2-12-e1107-s005.tiff]

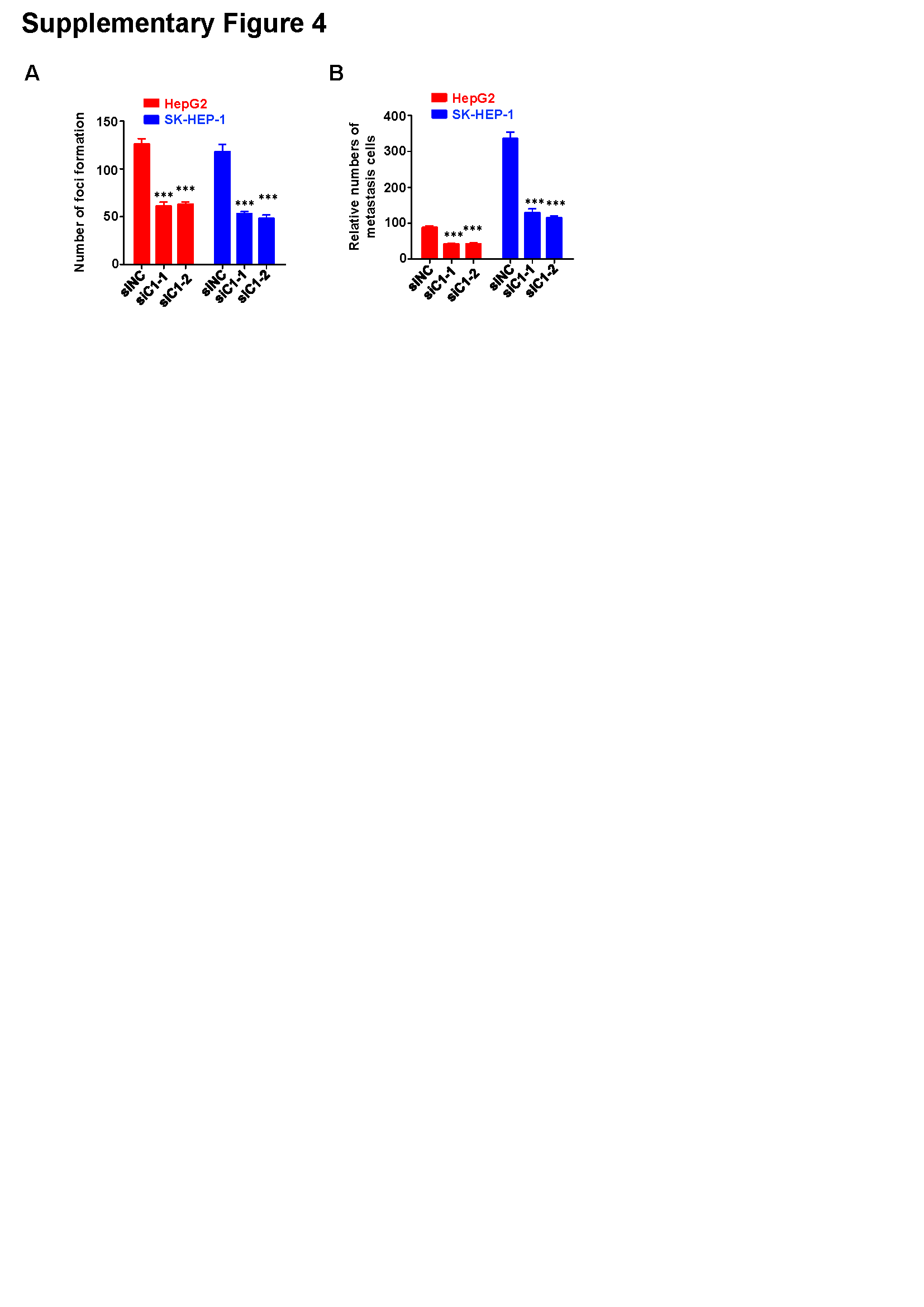

Supplement: Supplementary file 5 — Supporting Information [file CTM2-12-e1107-s006.tiff]

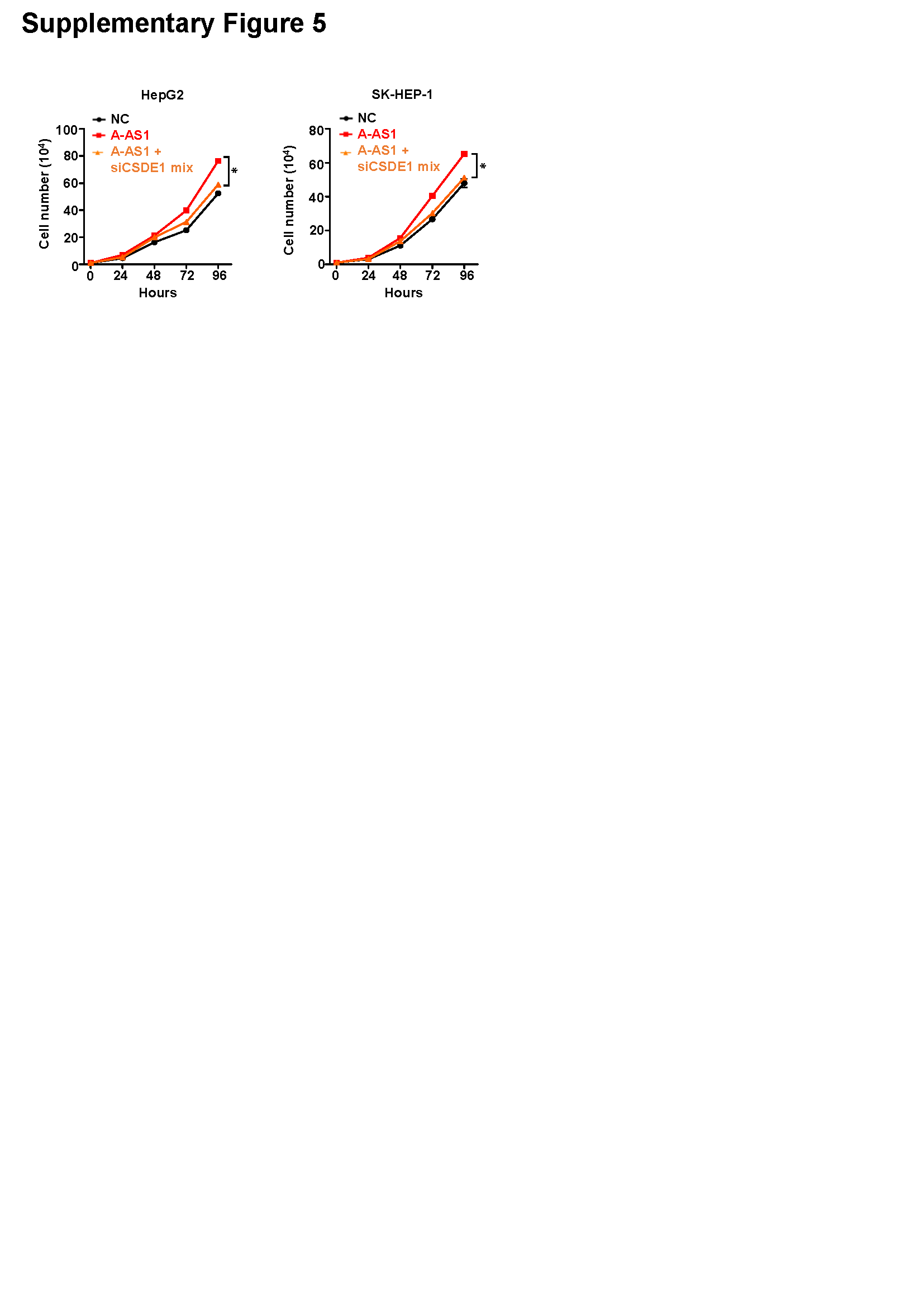

Supplement: Supplementary file 6 — Supporting Information [file CTM2-12-e1107-s007.tiff]
