# Supplementary material for: N6‐methyladenosine‐modified lncRNA ARHGAP5‐AS1 stabilises CSDE1 and coordinates oncogenic RNA regulons in hepatocellular carcinoma
Source: Clin Transl Med. 2022 Nov 10;12(11):e1107. doi: 10.1002/ctm2.1107 (PMC9647857; doi:10.1002/ctm2.1107)
Supplement: Supplementary file 7 — Supporting Information [file CTM2-12-e1107-s004.docx]

**Supplementary Figure legends**

**Supplementary Figure 1.** Relative expression levels of lncRNA ARHGAP5-AS1 in tissues and cell lines. (A) Increased levels of lncRNA ARHGAP5-AS1 were observed in resected HCC tissues, compared with paired normal tissues in multiple Chinese, Japanese and Italian HCC cohorts. (B) Relative expression of ARHGAP5-AS1 in HepG2 and SK-HEP-1 cell lines that stabilized either silenced *ARHGAP5-AS1* (by shRNAs) or overexpressed *ARHGAP5-AS1*. (C) The statistical analyses of colony formation assays. Data are shown as mean ± SD. **P* < 0.05, ***P* < 0.01, ****P* < 0.001 by unpaired Student’s *t* test.

**Supplementary Figure 2.** ARHGAP5-AS1 reduces migration and invasion capabilities of HCC cells. (A) In HepG2 and SK-HEP-1 cells, silencing of *ARHGAP5-AS1* inhibited wound-healing. (B) The stably enforced ARHGAP5-AS1 expression evidently accelerated wound-healing. (C,D) ARHGAP5-AS1 promoted invasion abilities of HepG2 and SK-HEP-1 cells. Cells on the lower surface of the chamber were stained by crystal violet.

**Supplementary Figure 3.** Immunofluorescence assays indicate that ARHGAP5-AS1 (red) and CSDE1 (green) colocalize in HCC cells.

**Supplementary Figure 4.** Silencing of *CSDE1* reduces proliferation and invasion capabilities of HCC cells. (A) The statistical analyses of colony formation assays. (B) The statistical analyses of transwell assays. Data are shown as mean ± SD. ****P* < 0.001 by unpaired Student’s *t* test.

**Supplementary Figure 5.** Silencing of *CSDE1* with siRNAs significantly inhibited proliferation of HepG2 and SK-HEP-1 cells with stably overexpressed *ARHGAP5-AS1*. Data are shown as mean ± SD. ****P* < 0.001 by unpaired Student’s *t* test.
